# Operationalizing Integrated Water Resource Management in Latin America: Insights from Application of the Freshwater Health Index

**DOI:** 10.1007/s00267-021-01446-1

**Published:** 2021-03-10

**Authors:** Maíra Ometto Bezerra, Derek Vollmer, Natalia Acero, Maria Clara Marques, Diego Restrepo, Eddy Mendoza, Bruno Coutinho, Ivo Encomenderos, Lina Zuluaga, Octavio Rodríguez, Kashif Shaad, Sarah Hauck, Ramon González, Francisco Hernandéz, Rodolfo Montelongo, Eliana Torres, Lina Serrano

**Affiliations:** 1 Conservation International, Moore Center for Science, Arlington, VA USA; 2 Conservation International Colombia, Bogotá, DC Colombia; 3 Conservation International Brazil, Rio de Janeiro, RJ Brazil; 4 Conservation International Perú, Lima, Perú; 5 Instituto Tecnológico de Monterrey, Centro del Agua para América Latina y Caribe, Monterrey, Mexico

**Keywords:** Integrated Water Resource Management, Water governance, Stakeholder engagement, Ecosystem services, Freshwater ecosystems, Latin America

## Abstract

Water crises in Latin America are more a consequence of poor management than resource scarcity. Addressing water management issues through better coordination, identification of problems and solutions, and agreement on common objectives to operationalize integrated water resources management (IWRM) could greatly improve water governance in the region. Composite indices have great potential to help overcome capacity and information challenges while supporting better IWRM. We applied one such index, the Freshwater Health Index (FHI) in three river basins in Latin America (Alto Mayo, Perú; Bogotá, Colombia; and Guandu, Brazil) to assess freshwater ecosystem vitality, ecosystem services, and the water governance system in place. The approach included convening management agencies, water utilities, planning authorities, local NGOs and industries, community groups and researchers to co-implement the FHI. The results provide detailed information on the ecological integrity of each basin and the sustainability of the ecosystem services being provided. All three basins show very low scores for governance and stakeholder engagement, thus improving both in the region should be a priority. The results also shed light on how the FHI framework can help inform decision-making to improve IWRM implementation by facilitating stakeholder engagement while contributing to coordination, identification of problems and solutions as well as agreement on common objectives. Because implementation of IWRM is part of the solution for the United Nations Sustainable Development Goal (SDG) 6.5 (“By 2030, implement IWRM at all levels, including through transboundary cooperation as appropriate”), our case studies can serve as examples to other Latin American countries to achieve SDG 6.5.

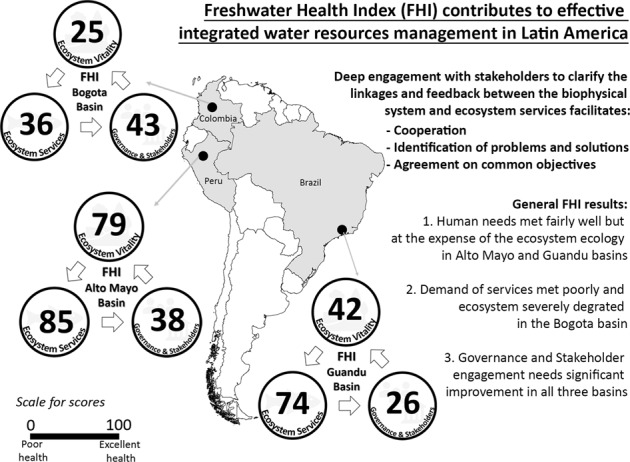

## Introduction

### Water Security in Latin America

Water crises in Latin America are more a consequence of poor management than resource scarcity, given the relative abundance of water in the region (UN WWAP 2015; Mattos et al. 2019). By receiving almost 30% of global rainfall and generating 33% of global runoff, water availability in Latin America is about 3000 m^3^ per capita per year—one of the highest per capita allocations of freshwater in the world. Nonetheless, pollution, economic inequities, and geographic location significantly restrict the provision of essential water-related services, such as water supply for human consumption. This situation underscores that the successful implementation of integrated water resource management (IWRM) to overcome water crises is still lagging in the region (Barbosa et al. 2017), despite the fact that IWRM-type instruments have been enshrined or ratified by many Latin American countries (Donoso and Bosch 2015; UN Environment 2018; Hidalgo-Toledo et al. 2019; Mancilla García et al. 2019).

Integrated Water Resource Management can be defined as:

A holistic approach that seeks to integrate the management of the physical environment within that of the broader socio-economic and political framework. The river basin approach seeks to focus on implementing IWRM principles on the basis of better coordination amongst operating and water management entities within a river basin, with a focus on allocating and delivering reliable water-dependent services in an equitable manner (UNESCO 2009, p. 2).

Actionable elements for effective implementation of IWRM derived from this definition include cooperation, identification of problems and solutions, and agreement on common objectives among water-related stakeholders (Mitchell and Hollick 1993; UN Environment 2018; Pahl-Wostl et al. 2020). However, Latin American countries face difficulties employing these three core IWRM elements.

Latin American countries report significant challenges in coordinating water management action across relevant stakeholder groups despite efforts to establish cooperation platforms such as river basin committees and commissions (Dourojeanni 2001; Akhmouch 2012; Guzmán-Arias and Calvo-Alvarado 2016). Part of the problem is due to an inability to communicate effectively across the wide range of stakeholders (Mancilla García and Bodin 2019) as technical information and knowledge need to be co-created in order to be broadly understood (Pahl-Wostl 2002; Wehn et al. 2017). This inability also compromises the identification of common problems and solutions as well as the agreement on common objectives, all further hindered by deficient technical capacity and data scarcity (Akhmouch 2012, UN Environment 2018). When stakeholders and managers have access to and can process richer datasets and co-create technical knowledge, they can participate more meaningfully in decision‐making negotiations, which, in turn, can lead to better-informed management (Feldman and Ingram 2009; Lemos et al. 2020). Conversely, a lack of knowledge and access to information often results in shortsighted and unsustainable use of water resources, as is often observed in Latin America (e.g., Empinotti et al. 2019; Deusdará-Leal et al. 2020).

Another critical reason for ineffective IWRM in Latin America is poor water governance (UN WWAP 2015; Grigg 2016; Espíndola and Ribeiro 2020), defined as the political, social, economic, and administrative systems in place that influence water use and management. In Latin America, inadequate water governance can manifest as unsustainable and informal water use practices, water pollution, and conflicts related to large infrastructure and natural resource development projects (Martín and Justo 2015). While adequate governance systems take time to establish, water governance can be improved in the short term by addressing coordination, identification of problems and solutions, and agreement on common objectives at the local management level (Grigg 2016).

To overcome these challenges, stakeholders can employ systematic and quantitative decision-support approaches that shed light on the relationship between the multiple dimensions of freshwater functioning and ecosystem services as well as the governance structure (Pahl-Wostl 2002; Pahl-Wostl et al. 2013; Pahl-Wostl et al. 2020). However, such approaches have not been broadly applied in Latin America, as management frameworks traditionally have been focused on assessing water quality and quantity (e.g., IANAS 2019) in a non-integrative fashion.

### Composite Indicators and the Freshwater Health Index

Composite indicators (or indices) have great potential to help overcome coordination, capacity, and information challenges while supporting better IWRM, because they summarize various measures of an ecosystem into fewer dimensions that can be easily communicated to a diverse audience (Heink and Kowarik 2010; Sutadian et al. 2016; Gibari et al. 2019). Indices are a popular method of communicating the overall health of aquatic environments to the wide range of stakeholders involved in their use and management (Dennison et al. 2007; Borja et al. 2008). They have been proven to maximize transparency and data-sharing as well as coordinate otherwise disparate stakeholders (Vollmer et al. 2018; McIntosh et al. 2019). Furthermore, indicators potentially enhance the rationality of policymaking and public debate by providing an objective, transparent, robust, and reliable information base, representing a state or trend over a given area and time period (Lehtonen 2015). However, in a review of more than 100 water-related indices, Vollmer et al. (2016) concluded that, in spite of the proliferation of water-related indices during the last two decades, it has been difficult for end-users to navigate and identify appropriate assessment methods for their informational needs and/or to understand and interpret results in a useful manner for decision-making. This suggests a need to not only better integrate environmental and socioeconomic indicators (Sullivan and Meigh 2007; Robinson et al. 2019; Laumann et al. 2019), but also engage with the end users and tailor indicators to the unique context in which they are being applied (Benavides et al. 2019; Bremer et al. 2020). This should contribute to more effective IWRM as watershed management needs to be grounded on issues and concerns that matter most in a particular place at a certain point in time (Watson et al. 2019).

The Freshwater Health Index (FHI) is one such approach, designed to quantify human water uses, impacts on freshwater ecosystems, and the governance system in place (Vollmer et al. 2018). Freshwater health, under this framework, is defined as “the ability to deliver water-related ecosystem services sustainably and equitably at the drainage-basin scale, linking the ecological function and condition of areas of service generation with communities of resource users”. It is implicit that sustainable and equitable long-term delivery of ecosystem services relies on long-term ecosystem function. Historically, a lack of practical support on how IWRM can make existing water planning, management and decision-making more rational, efficient and equitable has seriously limited the on-the-ground implementation of IWRM (Biswas 2004; Biswas 2008; McDonnell 2008). Therefore, by making clear connections between the different biophysical parameters characterizing the ecosystem and the degree to which these systems are providing the benefits people depend on, while being flexible enough to tailor to local and regional circumstances, the FHI framework has the potential to contribute to better operationalization of IWRM.

### Study Aim

The objective of this study was to apply the FHI in three basins in Latin America and analyze how the framework may help improve on-the-ground implementation of IWRM. Specifically, we examined how the FHI framework can: (1) contribute to better identification of problems and solutions, (2) coordinate and build agreement around common objectives; and (3) provide insights about the water governance system in the region. We also discuss how the FHI framework can facilitate stakeholder engagement in water resource management. As the FHI framework was developed and tested in large river basins in Asia (the Pearl River and Lower Mekong basins), this study also provides insights on how the framework can be adapted to differing contexts in smaller basins (<10,000 km^2^).

## Material and Methods

### Study Area

**Fig. 1:**
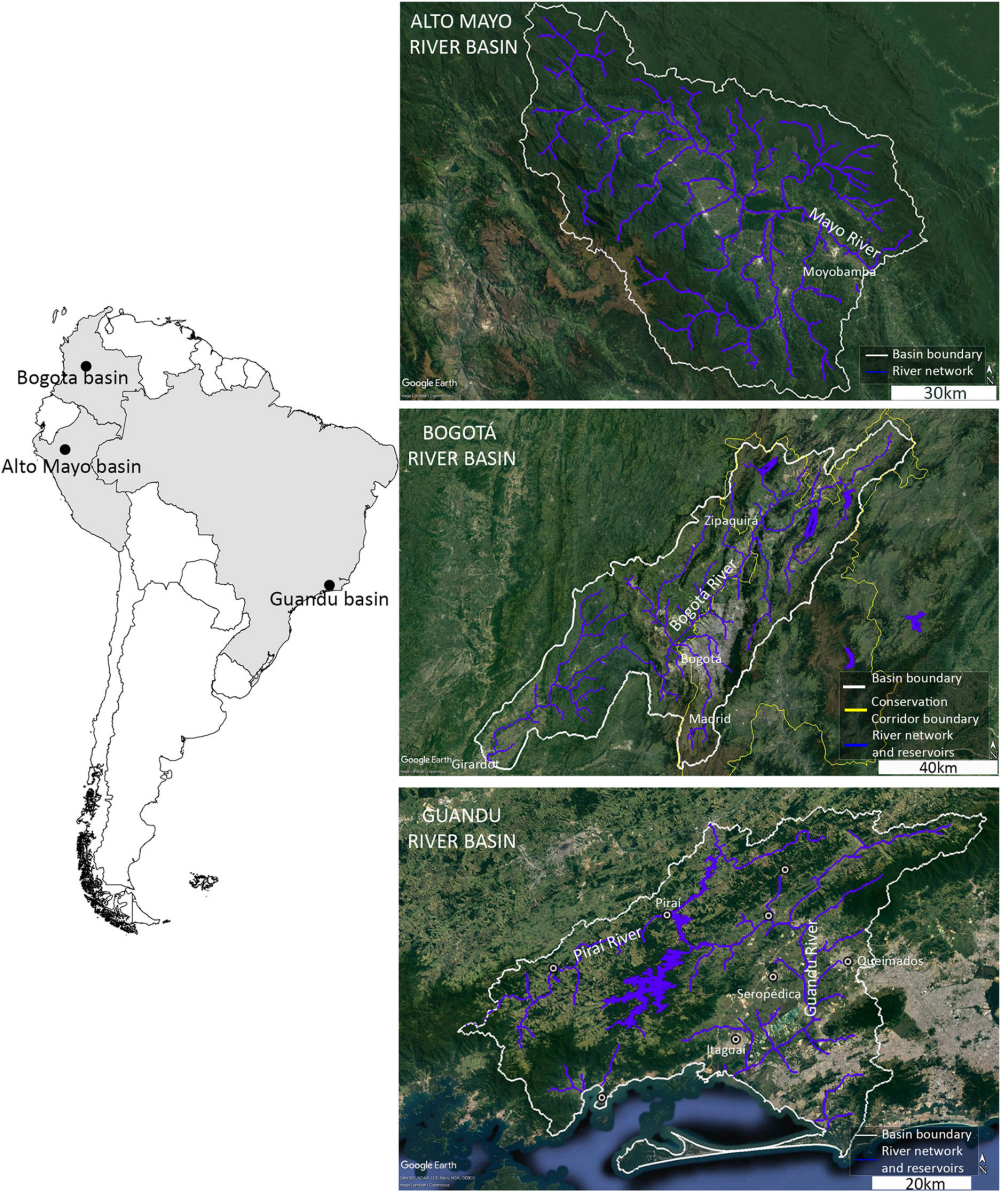
Study areas. Left: spatial location of study basins within country’s limits. Right: satellite imagine from Google Earth Pro for Alto Mayo River basin in Perú (top), Bogotá River basin in Colombia (middle), and Guandu River basin in Brazil (bottom). Basin boundary, major rivers and reservoirs are depicted

The FHI was applied in three basins in Latin America: Alto Mayo River basin, Perú; Bogotá River basin, Colombia; and Guandu River basin, Brazil (Fig. 1). Each basin represents a unique climatic, eco-hydrological, and sociopolitical context in the region.

#### Alto Mayo River basin, Perú

The Alto Mayo basin is located in the Andean-Amazonian foothills of Northern Perú, on the east side of the Andes and to the extreme west of the Amazon basin (between the coordinates: 5.24°S and 6.43°S latitude, and 77.46°W and 76.14°W longitude). The basin sits within the administrative region of San Martín, where the Mayo River forms the border between the provinces of Rioja and Moyobamba. The Mayo River, with an average discharge of about 99 m^3^/s, flows into the Huallaga River, which is a tributary of the Marañón River, which, in turn, flows into the Peruvian Amazon River. The natural vegetation in the region is characterized primarily by tropical moist broadleaf evergreen forest, of which 1820 km² is contained within the Alto Mayo Protected Forest. The region is home to roughly 248,000 people, including 14 Awajun indigenous communities with customary rights over about 20% of the basin area. The effective study area (7210 km^2^) includes the portion of the Mayo River valley under the jurisdiction of the Local Water Authority (ALA-Alto Mayo).

#### Bogotá River basin, Colombia

The Bogotá basin is located in the Eastern Cordillera of the Colombian Andes (between the coordinates: 4.25°N and 5.24°N latitude, and 73.53°W and 74.83°W longitude). It sits within the administrative region of Cundinamarca and includes the Bogotá metropolitan region, where approximately 10 million people reside (~25% of Colombia’s population). With a basin area of 5933 km^2^, the Bogotá River has an average discharge of about 79 m^3^/s and flows into the Magdalena River that drains to the Caribbean Sea. The natural vegetation in the region is characterized by various floristic physiognomies including Andean forest, shrubbery, and páramo vegetation (alpine-like wetlands). Much of the water provision to Bogotá city originates within the Conservation Corridor Chingaza-Sumapaz-Guerrero-Guacheneque, which not only includes the headwaters of the Bogotá basin but also encompasses the headwaters of four additional river basins (Sumapaz, Guavio, Guatiquía, and Guayuriba River basins). The Conservation Corridor has been designed to foster regional planning and environmental management to preserve the páramo and tropical montane cloud forest and, thus, guarantee Bogotá’s future water sustainability. Beginning in the early 1930s, dams were constructed in the Bogotá basin for water supply, resulting in seven reservoirs by 1994, with a total capacity of about 1150 million m^3^. As the population grew and urbanized, however, there has been substantial reliance on water from outside the Bogotá basin, and water transposition operations have brought in water from the Guatiquía and Guavio Rivers, both of which drain to the Orinoco River basin.

#### Guandu River basin, Brazil

The Guandu basin is located in the mountain and lowland areas of the southeastern Atlantic coast of Brazil (between the coordinates: 22.55°S and 23.19°S latitude, and 43.29°W and 44.28°W longitude). The natural vegetation in the basin includes various floristic physiognomies of the Atlantic Forest including ombrophilous forest, swamp forests, mangroves, and *restinga* (physiognomically distinct plant communities under marine and river-marine influence). The basin sits within the administrative region of Rio de Janeiro and is a highly engineered coastal watershed. Topographically, the Guandu basin would be composed of three major sub-basins (Guandu River basin—1385 km^2^, Guandu-Mirim River basin—190 km^2^, and Guarda River basin—346 km^2^) plus 30 smaller coastal catchments (501 km^2^). However, the administrative limit of the basin has been expanded to include an area where hydraulic modifications were made to reverse the direction of the Piraí river, which originally drained to the Paraíba do Sul River. As a result, Guandu’s official administrative area totals 3816 km^2^ and has an average discharge of about 150 m^3^/s. Increased water volume in the Guandu basin was intended to feed a sequence of hydroelectric power generation units integrated to the national electric energy grid system and to serve as the water supply system for 80% of the residents in the Rio de Janeiro metropolitan region (about 9 million people) and industries of national importance in the state of Rio (PROFILL 2017). In other words, the Guandu basin today can be described as a “intermediary basin”, serving as a conduit of water that originates in the neighboring basin, Paraíba do Sul, and whose water is consumed mostly outside of Guandu’s borders (Fig. S1).

### Freshwater Health Index

The FHI is a framework to assess the health of freshwater systems in their ecological and social dimensions, quantifying and mapping the multiple benefits that freshwater naturally provides (Vollmer et al. 2018). The FHI focuses on three main components: (1) Ecosystem Vitality (EV): the integrity and functioning of the ecosystem itself; (2) Ecosystem Services (ES): the benefits to people provided by a freshwater ecosystem; and (3) Governance and Stakeholders (GS): the structures and processes by which people make decisions related to water resources. Each of these three components is assessed with a suite of quantitative indicators and sub-indicators that are aggregated into an index. The Supplementary Material presents the description of methods used to calculate sub-indicators. Additional information on the FHI framework can be found at www.freshwaterhealthindex.org.

### Data Collection

For all three basins, quantitative information to evaluate the FHI sub-indicators came from a variety of sources, including in situ monitored water quality and discharge datasets, statistical yearbooks, soil erosion models, groundwater depletion maps, land cover maps, the IUCN Red List (IUCN 2018), and modeled hydrology. These were used to calculate sub-indicator values for the EV and ES components of the FHI. The sub-indicator scores range from 0 to 100, where 100 represents excellent health. Sub-indicators are aggregated into the indicator level and, subsequently, to the component level. Details on the actual datasets used to calculate each sub-indicator for each of the three study areas can be found in the Supplementary Material.

Values for the GS component sub-indicators were determined qualitatively via a survey using a Likert-type 5-point scale that was administered to stakeholders during workshops. The survey instrument, originally written in English, was translated into Spanish and Portuguese and then adapted (with the inclusion of local examples and expressions) by local experts. The survey includes 12 modules, each corresponding to a sub-indicator (Table 1), and each module contained 3–6 statements, totaling 51 statements. Survey respondents were instructed to only rate statements about which they felt knowledgeable. Responses were kept anonymous, although respondents’ sectoral affiliation (e.g., government, civil society organization) and geographic location (e.g., municipality) were recorded. In total, 22 respondents completed the survey for Guandu basin, 29 for Alto Mayo, and 60 for Bogotá. The larger sample size for Bogotá reflects the fact that in addition to stakeholders from the Bogotá basin itself, stakeholders from the other four major watersheds that form the Conservation Corridor mentioned above also attended the workshops and constituted about 50% of the total participants. Survey responses for each basin were averaged and normalized to give indicator scores on a 0–100 scale.

**Table 1:** Governance and Stakeholders indicators and sub-indicators adapted from Vollmer et al. (2018)

Major indicator	Definition major indicator	Sub-indicators
Enabling environment	It measures the constraints and opportunities that are enshrined by policies, regulations, market mechanisms, and social norms in governing and managing freshwater resources. It includes the extent to which typical water resource management functions (monitoring and coordination, planning and financing, developing and managing infrastructure, and resolving conflicts) are implemented through policies, institutions, management tools, financing, and accounting for various users and uses. It also considers the coherence of existing rights to resource use, including how water, land, and fishing rights are allocated, customary rights (including land tenure), and the degree to which these work in conjunction with formalized rights. Availability of different management instruments, as well as the capacity of skilled professionals working in water resource management fields, is also captured by this indicator	Water resource management
		Rights to resource use
		Incentives and regulations
		Financial capacity
		Technical capacity
Stakeholder engagement	It measures stakeholder interactions and the degree of transparency and accountability that govern these interactions. It assesses the access stakeholders have to information and data on local water resources in order to inform decision-making as well as the extent to which stakeholders have a voice within the cycle of policy, planning, and decision-making	Information access and knowledge
		Engagement in decision-making processes
Vision and adaptive governance	It measures the extent to which stakeholders engage in comprehensive strategic planning at the basin or sub-basin scale, the capacity to adapt to new information and changing conditions, and the existence of monitoring mechanisms to measure progress toward social and environmental objectives	Strategic planning and adaptive governance
		Monitoring and learning mechanisms
Effectiveness	It measures the degree to which laws are upheld and agreements are enforced, the distribution of water-related benefits, and the presence of water-related conflict	Enforcement and compliance
		Distribution of benefits from ecosystem services
		Water-related conflict

Stakeholders also assigned a weight to the indicators and sub-indicators of the ES and GS components using a two-level Analytic Hierarchy Process, calculated using a balanced scale in the BPMSG AHP online system (Goepel 2013). In this context, weights conveyed the importance stakeholders placed on aspects of governance and on which ES they considered more relevant in the basin. Weights were derived prior to any presentation of results so as not to potentially bias stakeholders’ perceptions about what is more or less important to them. The EV indicators were not weighted (i.e., left equal when aggregated) since their relative importance to freshwater ecosystems is considered to be objective and, therefore, should be informed by empirical rather than subjective means.

The derived weights were used to calculate the weighted geometric mean of the major indicator and component scores, so we performed a sensitivity analysis since weights are often an important source of uncertainty (Chen et al. 2013), particularly in group decision-making settings where individual priorities may differ. Using the principal component matrix from each individual’s AHP survey results, we calculated unique weights for each respondent. We then applied these weights when aggregating the major indicator and component scores for ES and GS, creating a unique set of scores based on an individual’s expressed preferences.

### Stakeholder Engagement

It has been widely recognized that stakeholder engagement is a principle for good water governance, because tackling the size and nature of water challenges requires a coordinated effort among those who play a role in decision-making as well as those affected by the actions and outcomes in the water sector (OECD 2015a). As such, stakeholder engagement is an integral part of the FHI framework and takes the form of a series of meetings aimed at involving stakeholders from a wide range of sectors (public, private, NGO and academia) acting at multiple levels (local, regional, and national) to co-implement the basin FHI assessment. For the applications in Latin America, seven stakeholder workshops (three in each basin and one joint meeting with stakeholders from all three basins) were convened over a period of approximately 12 months. The total number of stakeholders attending each meeting varied from 15 to 90 people, depending on the country (Table 2). In the Alto Mayo and Guandu basins, we worked closely with the respective watershed committee through bilateral meetings to reach out to the most relevant stakeholders and to deepen the discussions that occurred during the general stakeholder workshops. There was no formal organizing body for the Bogotá basin; thus, we selected and invited relevant stakeholders (~150) from a list of about 600 actors with whom Conservation International Colombia has been working for more than 10 years in the region.

**Table 2:** Description of the goals of each stakeholder meeting and characteristics of attendees in each meeting in each study basin

Meeting and main meeting goals	Basin	Meeting date	Total number of participants	Total number of institutions	Sectors represented
First meeting: introduction to FHI and surveys	Bogotá	06/20/18	24	15	4
	Guandu	05/14/18	33	25	4
	Alto Mayo	05/29/18	66	55	4
Second meeting: validation of preliminary results	Bogotá	08/16/18	79	54	4
	Guandu	09/20/18	29	15	4
	Alto Mayo	09/05/18	42	29	4
Third meeting: international exchange and use of FHI as decision-making tool	Bogotá	11/27–28/18	5	5	4
	Guandu		7	6	4
	Alto Mayo		7	6	3
Fourth meeting: presentation of final results and next steps	Bogotá	03/06/19	86	46	4
	Guandu	05/30/19	42	22	4
	Alto Mayo	03/13/19	31	17	4

Each meeting had different objectives, but all of them were linked to the broader FHI goals of: (1) providing opportunities for stakeholder interaction and coordination and (2) elucidating the relationships among the biophysical condition of the freshwater ecosystems, the benefits people receive from these ecosystems and the governance system in place. The specific goals of the first workshop in each basin were: to present the FHI framework, to administer the survey to populate the GS sub-indicators, and to administer the weighting exercise to evaluate the relative importance of indicators and sub-indicators in the ES and GS components. The main goals of the second workshop in each basin were to communicate and validate the preliminary results of the FHI and to assess the need to adapt or improve the calculation of any indicators. The third workshop, which brought together a subset of stakeholders from all three countries, was an opportunity for stakeholders to exchange their experiences and lessons learned throughout the FHI application process. The final workshop in each basin was held to share the results with a broader group of stakeholders, discuss those results in-depth, and identify next steps for action. Throughout the series of stakeholder workshops, the FHI methods were adapted to available existing data and then results were validated. Both of these steps (adaptation and validation) were guided by stakeholders’ experience and knowledge about the basin. As a result, the FHI baseline assessment produced for each basin can be considered to be a product co-implemented with stakeholders.

In addition, key technical stakeholders who were directly responsible for decision-making in the Alto Mayo and Guandu basins received personal training on the recently developed FHI desktop tool/calculator (https://www.freshwaterhealthindex.org/fhi-tool-download). This training provided another opportunity to ground the knowledge illuminated by the FHI assessment itself, which is essential for successful adoption of any approach (Vollmer et al. 2016; Wissen et al. 2016), and resulted in critical feedback and insights into policy relevance and potential management responses associated with the FHI results. Overall, the targeted stakeholder engagement throughout the FHI process allowed the identification of key management points from decision makers’ perspectives. Therefore, results and discussion focus on those topics, so that information generated by this study can be useful for more sustainable and integrated management of water resources in the region.

## Results and Discussion

### Overview

**Fig. 2:**
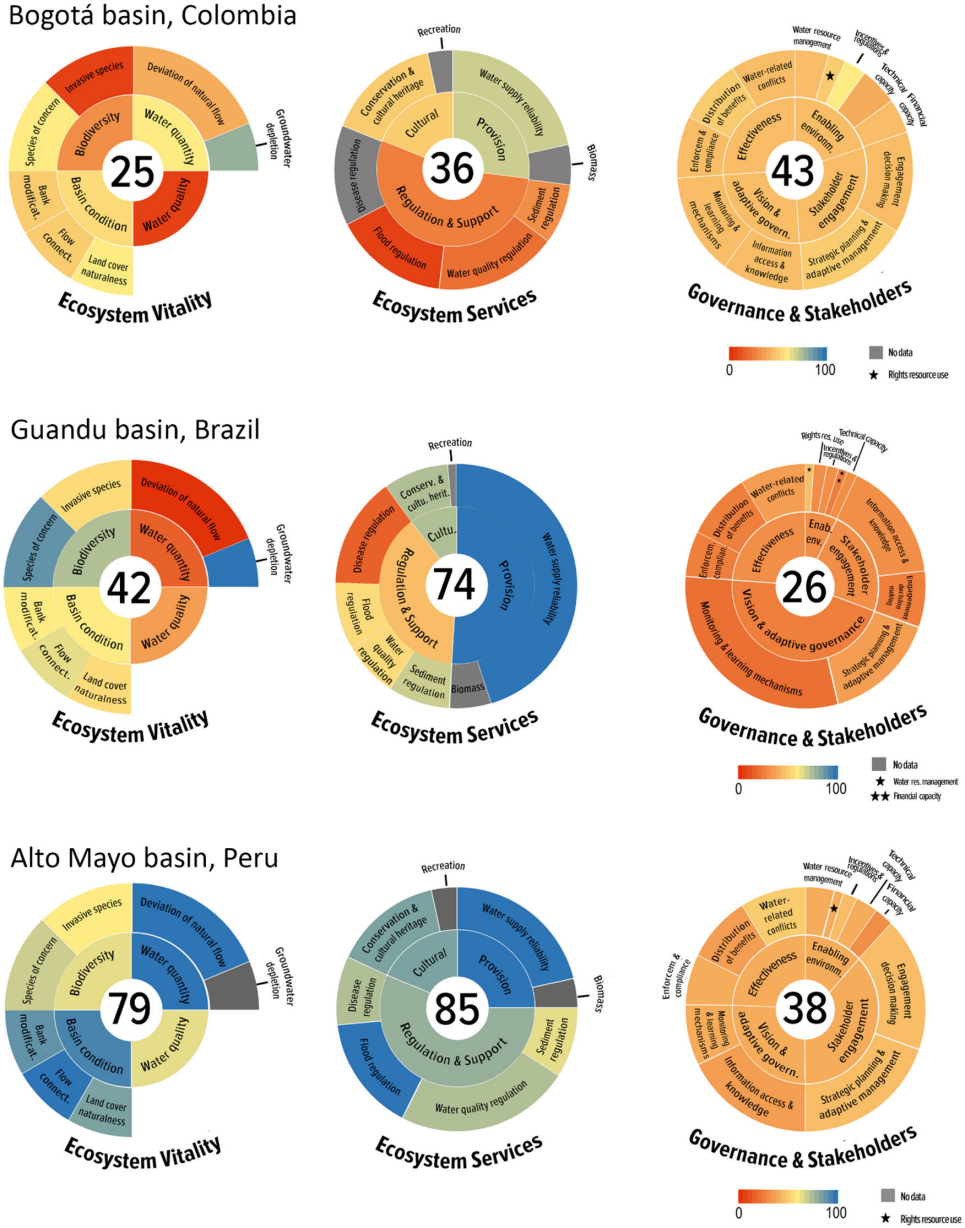
Freshwater Health Index (FHI) assessment for Bogotá River basin (top), Guandu River basin (middle), and Alto Mayo River basin (bottom). Number at the center of each circle indicates the aggregate final score for each FHI component (Ecosystem Vitality, Ecosystem Services, and Governance and Stakeholder) on a scale from 0−100, where 0 is poor health and 100 is excellent health. Inner circles represent major indicators and outter circles the sub-indicators. Scores were assigned a color based on the 0–100 gradient, and the size of each wedge for major- and sub-indicators of the Ecosystem Services and Governance and Stakeholders components reflects its relative weight determined through the AHP weighting exercise

The overall results for the Bogotá, Guandu, and Alto Mayo basins (Fig. 2 and Table 3) showed that human needs (as measured by ES) were being met fairly well in Guandu and Alto Mayo (ES_Guandu_ = 74 and ES_AltoMayo_ = 85); yet, was occurring at the expense of ecosystem ecology (as measured by EV; EV_Guandu_ = 42 and EV_AltoMayo_ = 79). In the Bogotá basin, which scored lower in both categories compared to the other two basins, the demand of water-related services was already being met poorly (ES_Bogotá_ = 36) and EV was found to be severely degraded (EV_Bogotá_ = 25). For the Governance and Stakeholders (GS) component, the summary scores highlighted that the governance structure may need to be significantly improved in all three basins (GS_AltoMayo_ = 38, GS_Bogotá_ = 43, GS_Guandu_ = 26) to secure ecosystem ecology while sustaining the benefits that people depend on.

**Table 3:** Result scores for the Freshwater Health Index sub-indicators for each of the three study basins

Major indicators	Sub-indicators	Bogotá	Guandu	Alto Mayo
		Scores		
*Ecosystem Vitality*				
Water quantity	Deviation of natural flow	35	4	98
	Groundwater storage depletion	77	93	*na*
Water quality	Water quality index	7	31	69
Drainage-basin condition	Bank modification (% of channel modification)	48	57	92
	Flow connectivity (Dendritic Connectivity Index)	50	64	100
	Land cover naturalness	57	53	82
Biodiversity	Species of concern	46	88	68
	Invasive species	10	50	60
*Ecosystem Services*				
Provisioning	Water supply reliability relative to demand	67	99	100
	Biomass for consumption	na	na	na
Regulating and support	Sediment regulation	26	67	60
	Water quality regulation	16	51	77
	Disease regulation	na	19	80
	Flood regulation	10	54	98
Cultural	Conservation/cultural heritage sites	49	73	83
	Recreation	na	na	na
*Governance and Stakeholder*				
Enabling environment	Water resource management	43	42	40
	Rights to resource use	48	26	36
	Incentives and regulations	57	29	49
	Financial capacity	40	20	30
	Technical capacity	39	27	39
Stakeholder engagement	Information access and knowledge	45	27	44
	Engagement in decision-making processes	43	22	37
Vision and adaptive governance	Strategic planning and adaptive governance	41	34	38
	Monitoring and learning mechanisms	42	19	34
Effectiveness	Enforcement and compliance	42	23	35
	Distribution of benefits from ecosystem services	46	23	31
	Water-related conflict	41	37	46

The preamble provided by the three components in the FHI framework (EV, ES, and GS) gives a quick overview of the overall trajectory of the freshwater systems. While the trade-off between ecological integrity and maximizing certain services is apparent by the negative correlations between the overall scores for the EV and ES components, clearly the three basins have specific contextual issues. Indicator scores helped to create a narrative in this context that can be useful for improving the planning process and support more informed decision-making, with the potential to lead to more effective IWRM. The paragraphs below highlight key management insights from interpreting the FHI results in light of the three actionable elements for effective implementation of IWRM: cooperation, identification of problems and solutions, and agreement on common objectives.

### FHI Contribution to Cooperation

In Latin America, the formation of river basin committees or commissions has been a common approach to facilitate cooperation around water resource management (Akhmouch 2012; Hidalgo-Toledo et al. 2019; Mancilla García and Bodin 2019; Trindade and Scheibe 2019). Participatory cooperation structures such as basin committees require effective communication and group understanding to coordinate stakeholders with different backgrounds, perspectives, goals, and interests. In other words, there is a need to foster social learning so that there is the generation of a broader knowledge from which collective decisions can be taken on the choice of priorities, measures, and strategies (Pahl-Wostl 2002; Wehn et al. 2017). Throughout the applications of the FHI in the three countries, many discussions, centered around the linkages between the components of EV and ES, appeared to contribute to the co-creation of knowledge, providing insights into the causes of a situation and the means of its possible transformation (Wehn et al. 2017).

**Fig. 3:**
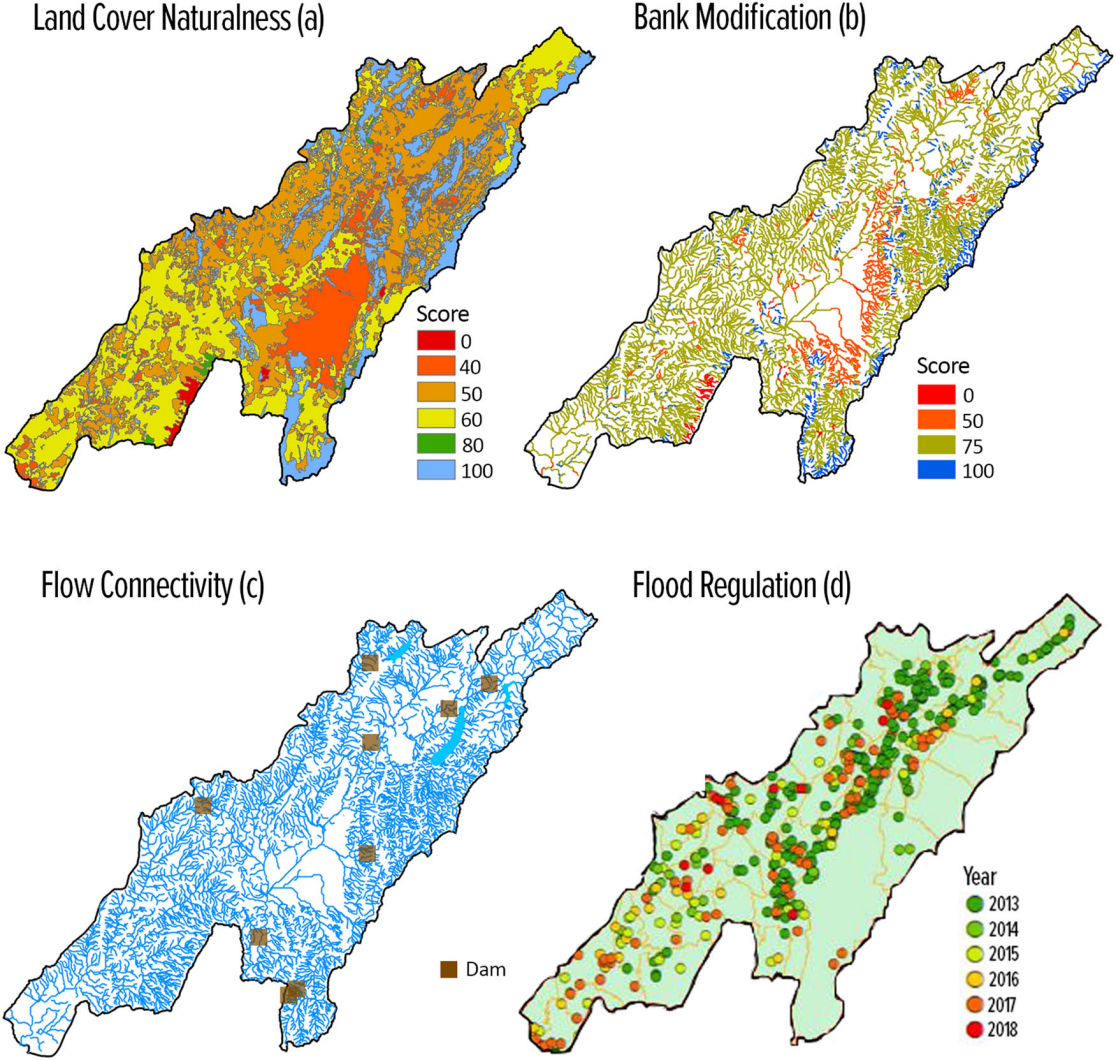
Results for the three sub-indicators of the indicator of Drainage-Basin Condition for the Bogotá River basin, Colombia: (**a**) Land Cover Naturalness - legend is the FHI score at the 30 m scale, (**b**) Bank Modification - legend is the FHI score for each stream reach analyzed, (**c**) Flow Connectivity - with the location of obstructions highlighted. And result for the (**d**) Flood Regulation sub-indicator of the indicator Regulating and Support Services - legend is the flood occurence for each listed year

One example of this happened during the validation of the preliminary FHI results in Bogotá. Through a series of group dynamics, stakeholders were able to collectively realize that the poor health of the Basin Condition indicator (51)—which is characterized by the status of the land cover, stream bank modification, and flow connectivity—can have clear negative consequences on Flood Regulation (10, Fig. 3d). While all three sub-indicators of Basin Condition were problematic in Bogotá (Table 3 and Fig. 3a–c), the moderate score for Land Cover Naturalness (57, Fig. 3a) was particularly useful to the co-creation of knowledge. Stakeholders connected their observations that the middle and upper portions of the Bogotá basin, where urbanization, industrialization, and farming (agriculture and pasture) concentrate, are more susceptible to frequent flooding; this, in turn, facilitated their recognition of the critical role that natural vegetation plays in flood regulation. As a result, stakeholders questioned the effectiveness of hydraulic engineering measures to control floods present in the middle and upper portions of the Bogotá basin. This analysis was further supported by the score of the Bank Modification sub-indicator (48, Fig. 3b), reflecting the high degree of channelization of the drainage system in urban- and agricultural-dominated areas. This example highlights that the FHI not only distilled information available in various technical reports (e.g., CAR 2006, 2019), but also provided a common language and a platform for stakeholders to discuss and absorb the information. The prospect is that such collective understanding of basin issues can enhance coordination of a wide range of stakeholders.

**Fig. 4:**
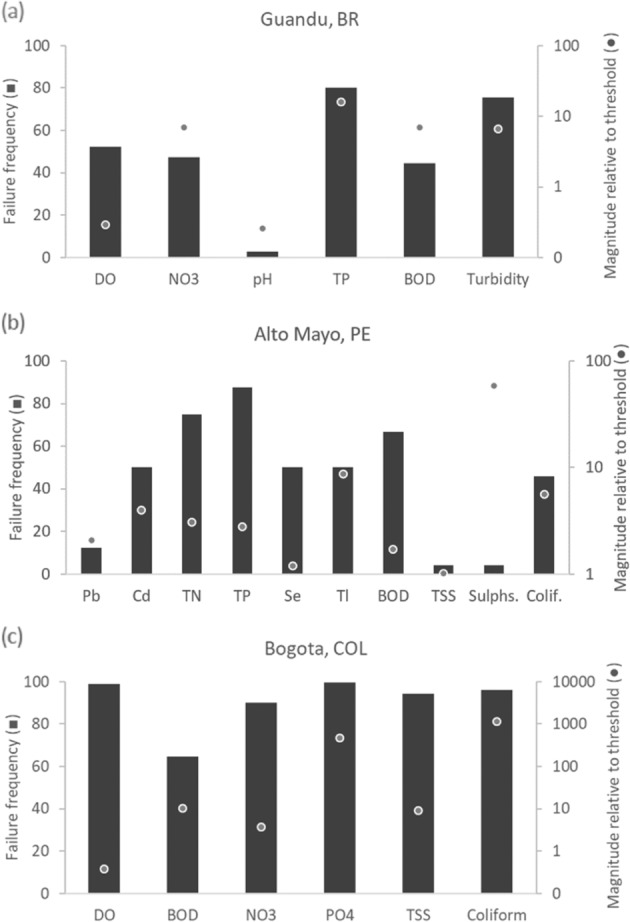
Results for the water quality indicator in the Ecosystem Vitality component for the study basins: (**a**) Guandu, (**b**) Alto Mayo, and (**c**) Bogotá. Left *Y* axis presents the percentage of times data failed to comply with threshold for each of the water quality parameter analyzed in each basin. Right *Y* axis presents the magnitude of failure in relation to the threshold on average (unit is the unit used for each specific parameter). DO is dissolved oxygen (mg.L^−1^), NO_3_ is nitrate (mg.L^−1^), TP is total phosphorus (mg.L^−^^1^), BOD is biological oxygen demand (mg.L^−1^), TN is total nitrogen (mg.L^−1^), TSS is total dissolved solids (mg.L^−1^), Sulphs. is sulfates (mg.L^−1^), Colif. is fecal coliforms (MPN/100 mL), PO_4_ is orthophosphate (mg.L^−1^), Turbidity (NTU), Pb is lead (μg.L^−1^), Cd is cadmium (mg.L^−1^), Se is selenium (mg.L^−1^), and Tl is thallium (mg.L^−1^). Not all parameters analyzed for Alto Mayo are presented in the graph

We also noted that using familiar topics as entry points often led to better cooperation around sensitive or typically neglected issues. For example, the relationship between water quality and disease regulation was a delicate topic during stakeholder meetings in all three countries. Stakeholders were aware that water quality affects both the ecological stream functioning (WQ_AltoMayo_ = 66, WQ_Bogotá_ = 7, WQ_Guandu_ = 31) as well as various human uses (WQR_AltoMayo_ = 77, WQR_Bogotá_ = 16, WQR_Guandu_ = 51). In most cases, where water quality appeared compromised, the realization that sewage contamination (Fig. 4) was the main driver of these patterns served as a stimulus for discussions about the occurrence of water-related diseases. This dynamic was most noticeable by the results for Guandu.

**Fig. 5:**
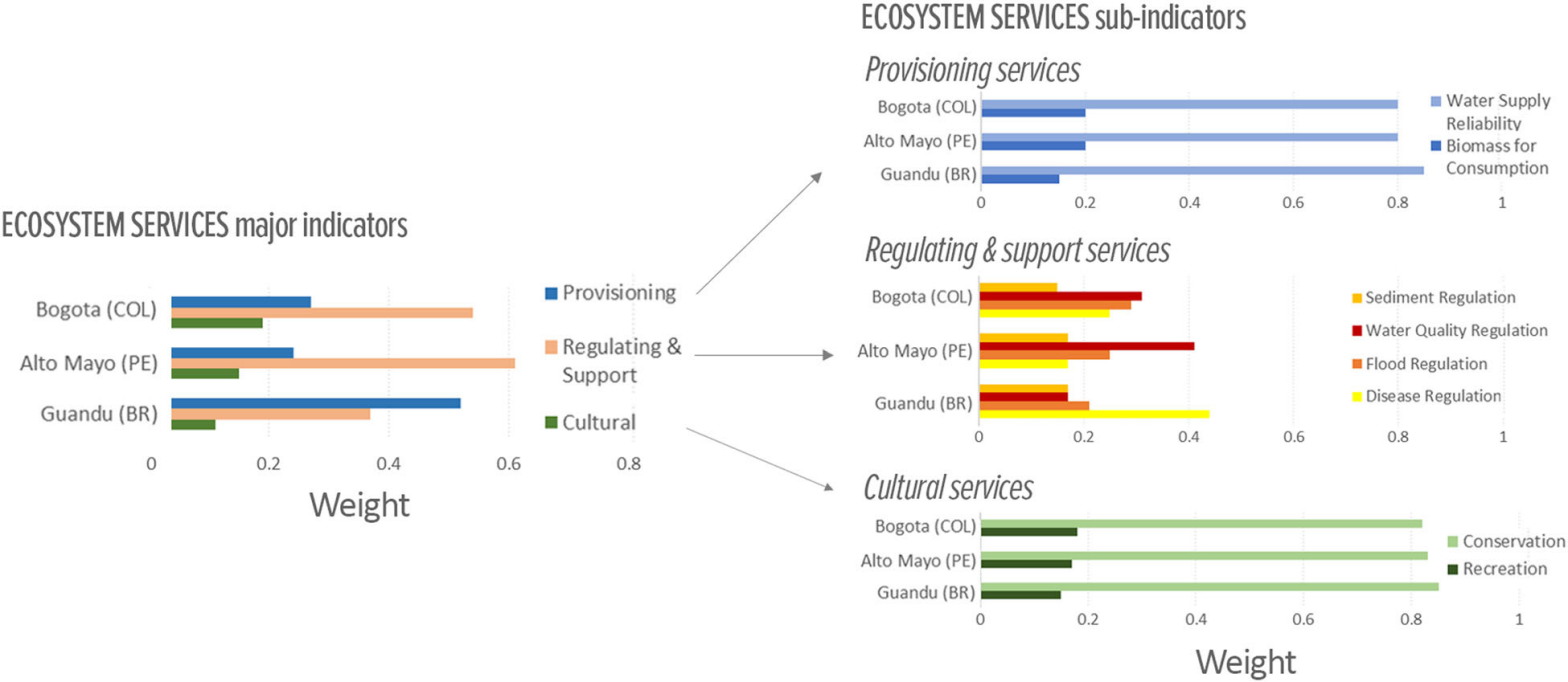
Results for the weighting exercise assessing the relative importance stakeholders give to different ecosystem services for each study basin. Panels show results for the major Ecosystem Services indicators (left) and sub-indicators (right)

Water-related disease in Guandu was a major issue for stakeholders given that they assigned it the highest relative importance among all four regulating services (Fig. 5) and its score (19) was the second lowest among all measured ES indicators (Table 3). The low score for Disease Regulation, which included scores for four different diseases (see “Methods” in Supplementary Material), was driven by the individual score obtained for diarrhea (0.12) that was calculated based on levels of fecal coliforms. The results showed that 19 of the 28 monitoring stations had levels exceeding Brazil’s Class 1 standard of 1000/100 mL more than 15% of the time.

Poor water quality and its relationship with water security has been a chronic problem in most developing countries mainly due to discharges of raw sewage into rivers and streams (Benavides et al. 2019; IANAS 2019). Although this potential linkage seems intuitive, disease and public health issues are not typically addressed directly in water resource management discussions. By assigning a high importance to Disease Regulation in relation to other regulating services and noting its poor performance (Fig. 5), stakeholders underscored the need and their willingness for better coordination particularly since this issue of disease burden is also closely associated with issues of social justice; another priority that is implicit but not commonly operationalized in IWRM.

### FHI Contribution to Identification of Problems and Solutions

By representing freshwater ecosystems as dynamic social–ecological networks, we observed that the FHI can contribute to deepening managers’ ability to understand linkages and feedbacks between human water needs and the basin’s ecological condition and ability to meet those needs, ultimately improving their capacity to identify solutions. The sediment regulation indicator results in Alto Mayo demonstrate how connections between the status of certain ES and the expectation stakeholders have for the supply of those services can reveal hidden problems or “blind spots”.

Sediment Regulation had the lowest score (60) among all measured services in the Alto Mayo basin, indicating that its demand is being met moderately, an outlier in comparison to all the other services (with scores > 75). Despite the relatively low score, Sediment Regulation was the service for which stakeholders showed the least concern, based on its low weight (Fig. 5). This mismatch between low score (e.g., signaling a problematic area) and low weight sparked inquiries from stakeholders during meetings, which created the opportunity to discuss related management topics, such as hydropower expansion coupled with forest conservation.

Six hydroelectric power plants have been authorized in the Alto Mayo basin: two within the borders of the study area (CH Las Orquideas and CH Naranjos II) and four in the Mayo River just downstream of the study basin outlet (CH Mayo I-IV) (ANA 2015a, b). Such hydropower expansion is part of the plan to increase Peru’s energy capacity, since only 1% of the surface water volume of the Huallaga basin, which encompasses the Alto Mayo basin, is used for human activities (ANA 2016). At the same time, the Alto Mayo basin is home to one of the most important forest reserves in the country. However, the pattern of continued deforestation in the region and its association with increased soil erosion may compromise hydroelectricity generation capacity in the long term.

Linking these issues through the FHI provided an opportunity for stakeholders to understand the importance of forests for sediment retention and the relevance of conservation-based territorial plans to reduce erosion while safeguarding the provision of hydroelectric energy. These issues were not under discussion by the Alto Mayo sub-basin committee, which previously had focused almost exclusively on water quality and quantity. In addition to elucidating priorities, this example also shows that viewing socio-ecological linkages and feedbacks can facilitate understanding of where and to what extent water-related ecosystems support services. The expectation is, therefore, that such clarity could serve as a strong motivation for stakeholders to try to minimize negative impacts and protect upstream ecosystems as part of the solution.

### FHI Contribution to Agreement on Common Objectives

Agreement is key for effective IWRM as it can seal the willingness of a diverse group of stakeholders to attain common objectives. Agreements contribute to planning and preparedness around watershed management issues such as the development of water infrastructure and placement of protected areas (e.g., Jiménez et al. 2020). Further, the process of building agreements may help establish or maintain important channels of communication for stakeholders that may have less than ideal diplomatic relationships but still need to work together on basic issues related to local and regional water security (Jiménez et al. 2020). For instance, in highly engineered basins, such as the Guandu and Bogotá basins, there is commonly a power imbalance between water utility companies and other water users, but a there is still a common objective of providing water of sufficient quantity and quality to the large metropolitan populations.

Consensus building is a key first step to achieving agreement, and the application of the FHI in Latin America was effective in attaining it. The emergence of a shared vision during the final stakeholder meeting in Guandu is a practical example. Stakeholders in Guandu collectively agreed that the basin’s management must aim for water resilience within the basin, which means a decreased reliance on water diversion from the Paraíba do Sul basin. This common vision emerged from reflections on the meaning and status of two indicators, Water Supply Reliability Relative to Demand (WSRD) and Deviation from Natural Flow (DvNF).

**Fig. 6:**
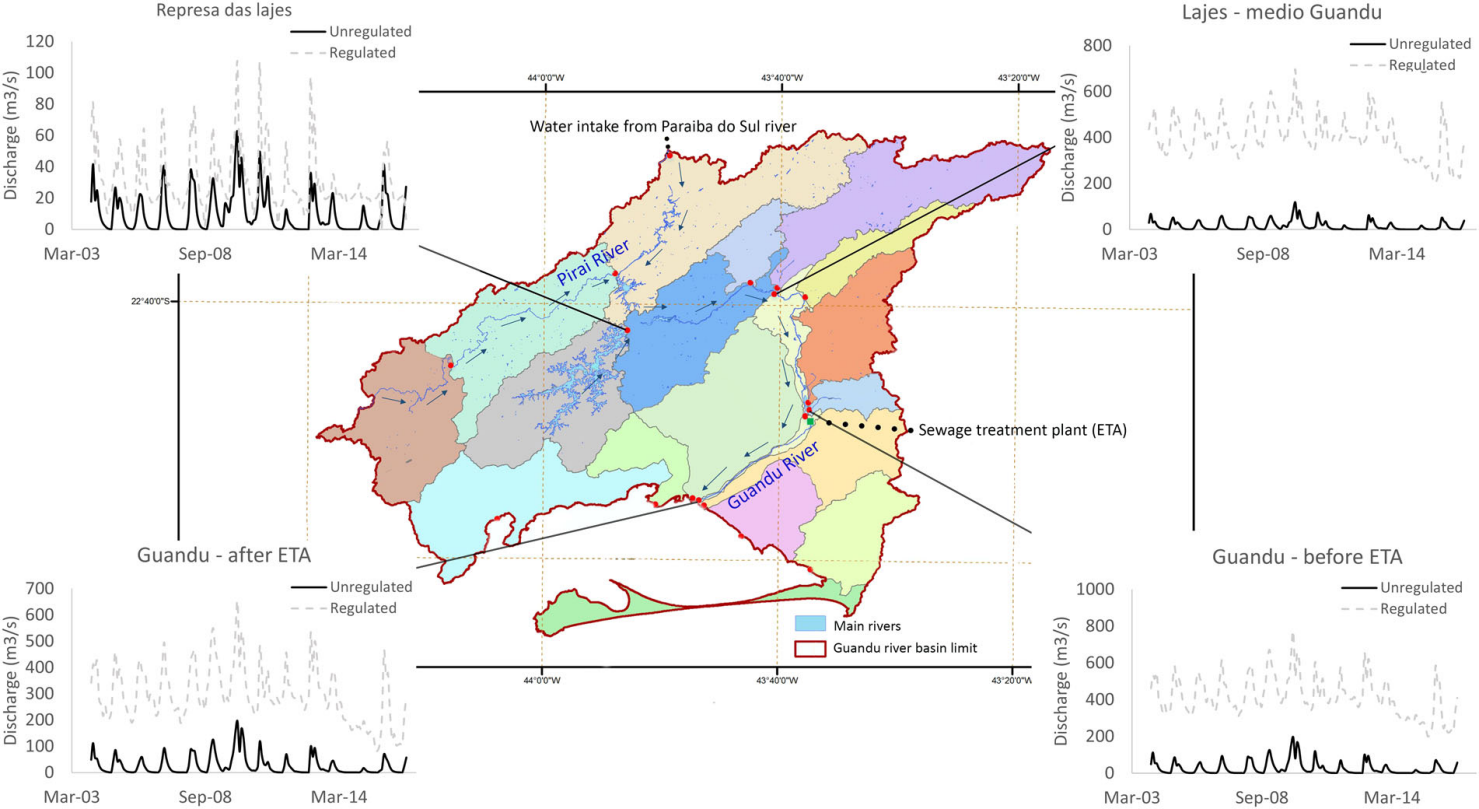
Representation of the modified flow of the Guandu basin, Brazil. Arrows indicate rivers’ flow direction and highlight the inverted flow of the Piraí river (North of the basin). The colors describe each sub-basin included in the hydrological model and the red dots locate the outlet of each sub-basin. The graphs exemplify the comparison between regulated discharge under current conditions and modeled unregulated discharge for four sub-basins for the period January 2004 - December 2016

First, stakeholders considered that the high value of WSRD (99), indicating a near perfect balance between water demand and supply, can mislead management actions because it does not depict Guandu’s natural water availability, which is far from sufficient to meet the Rio de Janeiro Metropolitan Region’s water demand without the transposition from the Paraíba do Sul that provides about 90% of the total water volume in Guandu (González-Bravo et al. 2019). In other words, the high WSRD score merely reflects that water needs were almost universally met, regardless of the source. Yet, stakeholders recognized that the results highlight how fragile water availability is in the region because the score of WSRD must be analyzed alongside the score of DvNF. As the very low DvNF score (4) is associated with significant increases in discharge from water transfers from the Paraíba do Sul basin (Fig. 6), there is a constant threat of water shortage because the Paraíba do Sul also supplies water to the largest metropolitan region in Brazil (in São Paulo) (ANA 2015a, b; PROFILL 2017).

The common vision of a resilient Guandu basin was corroborated by results from the weighting exercise, indicating that the ES that stakeholders were most concerned about was water provisioning. Provisioning services were valued much higher than the other two groups of services (Fig. 5, weight of 0.52). And, between the two provisioning services sub-indicators, Water Supply Reliability was by far the more important service (0.85) compared to Biomass Consumption (i.e., fish and related aquatic material). Moreover, the measure of consensus for this weighting was 0.93 (1.0 being the maximum), which was the highest degree of group consensus we observed across all three basins and all indicator groupings (Vollmer et al. 2020). Thus, the FHI results provided a platform for building consensus around a common vision that is fundamental for achieving further agreement on common problems. And it is clear from this example that, more than sophisticated data or models, consensus on management issues can be built via deep discussion of more basic information together with the end-users.

### Governance Results

The Governance and Stakeholder (GS) assessments were the first quantitative assessments of water governance undertaken at the basin scale for the three study areas and among the first examples of this level of analysis of water governance within Latin America (Vollmer et al. 2020). The aim of the GS component is not to provide objectively “correct” scores but to illuminate stakeholders’ perception of the effectiveness of the GS in place as it relates to freshwater management and use. Compared to the other two FHI components (EV and ES), the low score for the GS component was one of the most sensitive subjects discussed during stakeholder meetings in all three basins.

On the one hand, as the scores reflected the collective perception among stakeholders from each basin, the discussions created a space for frank dialog and reflection on sensitive governance issues. An environment of honesty and trust such as that fostered during the FHI meetings is required to build public confidence and ensure inclusiveness of stakeholders, thereby safeguarding a favorable environment for good water governance according to the OECD Principles on Water Governance (OECD 2015b, 2018). On the other hand, the low score for GS compared to the other two FHI components (Fig. 2) reflects the continued global challenge of achieving effective water governance (Neto et al. 2018; Romano and Akhmouch 2019) and emphasizes the enormous improvements in a wide range of social areas (Tables 1 and 3) required to attain effective watershed management in face of the rapidly changing conditions that will make water governance even more complex in the coming years.

When reflecting on the comparative GS scores across basins, stakeholders from Brazil offered the following insight: as GS scores are based on perception, they are likely anchored by stakeholders’ expectations, and although this varies by individual, it may also be generally influenced by the “maturity” of the water governance system in each place. This reflection is partially corroborated by the fact that the lowest GS score was observed for Guandu, followed by the GS score in Perú, and Colombia with the highest GS score (Table 3 and Fig. 2). Guandu belongs to the country (Brazil) with the oldest water law (1997) among the three countries studied—the water law in Perú and Colombia is more recent, 2009 and 2010, respectively. This difference of “maturity” in the structuring of water resources management could be influencing the expectation that stakeholders in each basin have about the effectiveness of their respective water governance system. In other words, the lowest observed GS score for the Guandu basin in relation to the scores of the other two countries could be reflecting the fact that stakeholders in Guandu are demanding and expecting more from their GS at the moment—a pattern also observed in the FHI application in the Lower Mekong basin, when comparing Vietnam, Cambodia, and Laos (Liu et al. 2019). A more mature water governance in Brazil given its older water law is possible because better understanding on the role of a collaborative and democratic management models, such as watershed committees, are observed where stakeholders participate on committees that are older, and that have management instruments under implementation and greater institutional articulation (Trindade and Scheibe 2019). Still, the pattern described above underscores that the governance indicators of the FHI are not designed to be compared across countries, given the differing contexts as well as stakeholders’ expectations.

**Fig. 7:**
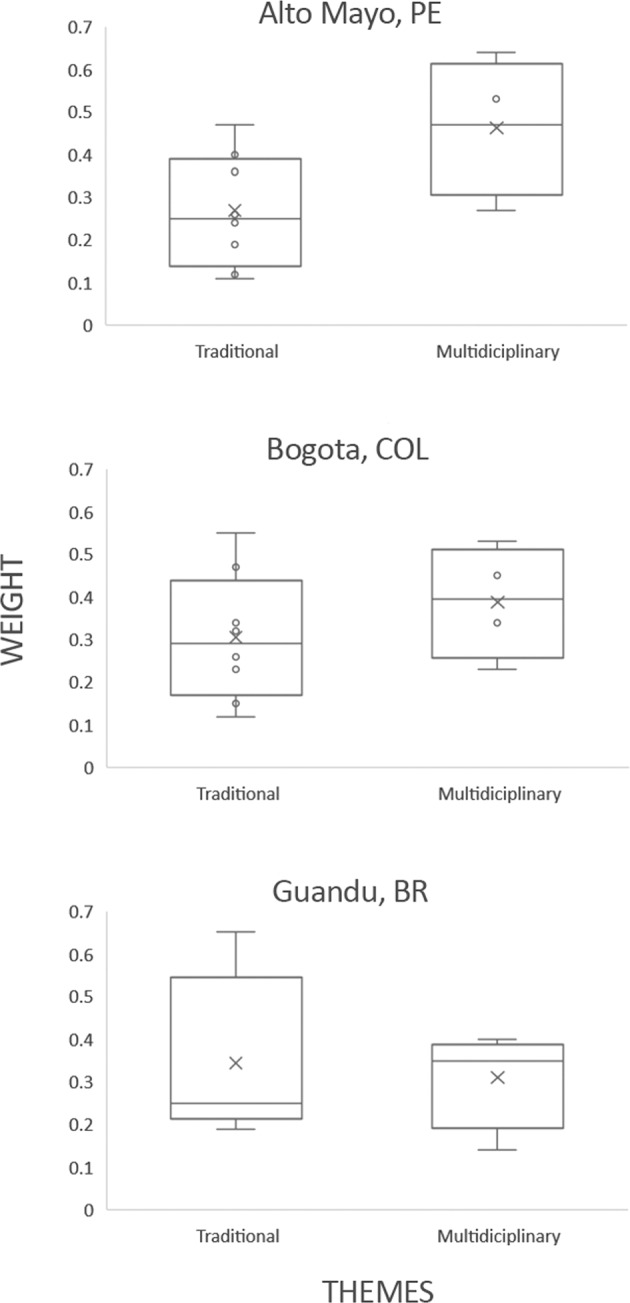
Weight distribution for the Governance and Stakeholder (GS) sub-indicators considered to measure: (1) traditional governance themes (FHI GS sub-indicator list in this category includes: finance and technical capacity, law enforcement, water-related conflicts, incentives and regulations, rights to resource use, information access and knowledge, monitoring and learning mechanisms, and enforcement and compliance); and (2) multidisciplinary governance themes (FHI GS sub-indicator list in this category includes: strategic planning and adaptive governance, distribution of benefits from ecosystem services, engagement in decision-making processes, and water resource management) for each study basin. The box-and-whisker plots show the minimum, first quartile, median, third quartile, and maximum of the set of weights for each category

One of the requirements for effective water governance is transdisciplinary thinking, so that decisions adequately balance competing priorities and account for the long-term sustainability of freshwater ecosystems (e.g., Pahl-Wostl et al. 2020). Specific analysis on whether stakeholders were predisposed to transdisciplinary thinking suggests that stakeholders from Bogotá and Guandu might be less focused on cross-sectoral and interdisciplinary approaches to governance, compared to those of the Alto Mayo. This is because stakeholders in the Alto Mayo basin tended to grade interdisciplinary themes higher than stakeholders in the Bogotá and Guandu basins (Fig. 7). Such a pattern aligns with the fact that, in most large, urbanized basins, such as Guandu and Bogotá, water utilities and large infrastructure operators typically have formidable ability to exercise social and political dominion over the management of water resources due to, for example, their large-scale, centralized infrastructure (Swyngedouw 2009). As a result, technocratic thinking usually prevails (Trindade and Scheibe 2019; Lemos et al. 2020). In the Alto Mayo basin, conversely, stakeholders tended to think in a more integrated and collaborative way, possibly because the basin is still in a near-pristine condition, i.e., the connection that residents hold with nature in the basin might be allowing for a more holistic view of water management. This is corroborated by the fact that the water management system in Perú was inspired by principles of IWRM, such as promoting a holistic vision of water accounting for different interests (Mancilla García and Bodin 2019). Interestingly, the pattern found for Alto Mayo somewhat contradicts the fact that participation in water councils in Perú tend to be highly technocratic as stakeholders there present the identity of the councils as very much tied to the technical secretariat, staffed with engineers from the National Water Authority, who usually prepare and orient the discussions at council meetings (Mancilla García and Bodin 2019). Overall, there is still a need to foster transdisciplinary thinking in a more direct manner to improve IWRM practice in Latin America. And the FHI framework can contribute by bringing together and providing a common language for a diverse group of stakeholders that would otherwise may not be working collaboratively.

### Considerations on the Results of Weights

The sensitivity analysis we performed on the individual weights (Fig. S2) did underscore that not all participants agreed on the relative priorities. While this does not impact the scores for the 18 sub-indicators measured under the ES and GS components, it would have an impact on the aggregated scores for the seven major indicators, as well as the top-level component scores. We found that the range of possible scores was much greater for ES in all three basins, and much more constrained for GS, due mainly to the fact that the GS sub-indicators had uniformly low scores (Table 3), and thus strong preferences for one over another had limited effect on the weighted mean. The impact of varing the weights was most pronounced in Guandu, where, at an individual respondent level, the score for the ES component ranged from the mid-40s (e.g., critically unhealthy) to 90, but this variation was determined mostly by the relative importance an individual placed on the Regulating and Support Services sub-indicators (which had scores ranging from 19 to 67). As a group, Regulating and Support Services also scored far below Provisioning and Cultural Services, leading to another source of uncertainty when the three major indicator scores were averaged to the ES component score. In other words, while the group average weighted score of 74 suggested moderate health, there are individuals within the group of decision makers who would likely disagree with that generalization.

### Insights on Information Needs

An inability to generate and process data to create the transdisciplinary knowledge required for IWRM has been previously identified as one of the most important challenges Latin American countries must overcome to improve water governance in the region (Akhmouch 2012) and is still a major bottleneck to be overcome (Benavides et al. 2019). These deficiencies were observed in all three study basins in the form of data scarcity and fragmentation (e.g., multiple institutions holding different portions of the same dataset) as well as low-quality data (e.g., incomplete timeseries and datasets lacking metadata). Not surprisingly, stakeholders in all three basins perceived Technical Capacity as one of the most critical sub-indicators in the GS component (Table 3). Many participants expressed particular interest in platforms to aggregate a multitude of datasets and visualize the relationships between different variables in a meaningful and simple way. Enhanced technical capacity not only can contribute to better-informed decisions (Lemos et al. 2020) but also can contribute to the democratization of decision-making. In Brazil, for example, the lack of knowledge of discussion topics by representatives of civil society is one of the main reasons for the sporadic and limited participation of this group of users in watershed committees (Trindade and Scheibe 2019). All of this strengthens the case for the utility of the FHI framework to integrate available data as well as co-implement and disseminate knowledge on watershed management.

Not only can the FHI contribute to improving access to useful information, but it can also identify critical data gaps that, if filled, could contribute to better watershed management locally. Following the global pattern (Foster and Ait-Kadi 2012, Lall et al. 2020), one significant gap identified in all three basins was groundwater data, as no organized monitoring scheme appears to have been implemented for the study basins. This is mostly notable in places such as Alto Mayo because, although stakeholders rely primarily on surface water allocation to meet their needs, there are plans to expand groundwater abstraction in the near future (ANA 2015a, b). As the Alto Mayo basin is home to one of the most important groundwater sources from karst formations in South America (Grandjouan et al. 2017) due to its high resurgence rate (discharges can reach up to 24 m^3^/s) (Bigot et al. 2014), filling the knowledge gap on groundwater availability and extraction is critical for the sustainable use of groundwater resources in the region.

Another data gap of relevance to watershed management observed in all three study basins was data needed to calculate the indicator of Biomass for Consumption. Inland fisheries are commonly afforded low importance among decision makers, despite their relevance as a food source (Lynch et al. 2020). Although inland extractive fishing appeared not economically relevant for any of the three basins, artisanal fishing might be for local communities. Still, understanding the extent of its significance requires improved monitoring systems as exemplified by the situation in Guandu. There, fishing activities in the Sepetiba bay, into which the Guandu River flows, are one of the main sources of fisheries products in the region (Brasil 2013), but they have been threatened directly by the management of the Guandu basin in the form of increased heavy metals discharge, predatory fishing, and lack of enforcement in the region (Ribeiro et al. 2014, 2015; MPF 2015). For FHI assessments related to coastal basins, therefore, data on marine fish species that can enter freshwater rivers and streams, such as the *Diapterus rhombeus* in Guandu, are crucial to providing a more complete understanding of the relationship between people’s dependence on biodiversity and the overall state of freshwater health.

## Conclusion

Although the importance of IWRM to solve the water crises in Latin America has been widely recognized, approaches that can truly operationalize IWRM by analyzing the interplay between governance, stakeholders, freshwater ecosystems and their services have rarely been applied as part of the solution. This study explored how the FHI can contribute to more effective and efficient on-the-ground IWRM by being a platform for better coordination, identification of problems and solutions, and capacity to build agreements.

With applications in three basins, the FHI was shown to not only facilitate the operationalization of those three key elements but also elucidate the linkages, feedback, and synergies between the ecological condition of the basin and the benefits it provides to people across distinct settings. Dedicated stakeholder engagement helped the different groups of actors understand these relationships between EV and ES in a meaningful way. This highlights the power of social–ecological approaches to foster better communication and cooperation among stakeholders, which ultimately can lead to consensus around management priorities and the long-term environmental sustainability of a basin. Moreover, as implementation of IWRM is one of the elements that must be reported on for the United Nation’s SDG 6 (UN 2015), the linkages between FHI and IWRM discussed in this study could serve as examples for Latin America as a whole to improve IWRM, in furtherance of achieving specific targets under SDG 6.

An additional value of the FHI framework is its contribution to overcoming technical and information gaps that are currently some of the most relevant factors hindering effective water governance and IWRM. The application in three different basins showed that a complete overview of the basin’s health can be obtained very quickly with available secondary data for a variety of contexts, and also brought attention to critical data gaps or deficiencies. Assessments like this might be the only source of comprehensive knowledge to tackle water resource issues in countries where the political–administrative structure for water management, informational capacity, and finances are limited.

The applications in Latin America also highlighted the plasticity of the FHI tool to adapt to varying degrees of data availability. Its common platform, simplicity, shared terminology, and comparability of indicators and targets provided a “common language” for multiple institutions to engage in the complex interrelated topics of water resource management. This addresses the fact that IWRM cannot be implemented in a standardized fashion; instead, it must be flexible enough to consider contextual conditions and differences in beliefs, attitudes, norms, etc. (Watson et al. 2019).

Finally, water crises are a global issue (Hofste et al. 2019) that, to be solved, require context-based solutions because water is inseparably linked to watersheds and ecosystems (which dictate where, and how much water flows) with a mix of private and public users; making it a resource difficult to manage with a “one-size-fits-all” approach (Vollmer and Harrison 2021). With this study, we have provided clear guidance on how to make such context-based analyses actionable, and our methods can be applied at a variety of scales and situations around the world.

## Supplementary information

Supplementary Material

## Data Availability

Software applied: Freshwater Health Index.
