# Supplementary material for: Operationalizing Integrated Water Resource Management in Latin America: Insights from Application of the Freshwater Health Index
Source: Environ Manage. 2021 Mar 10;69(4):815–34. doi: 10.1007/s00267-021-01446-1 (PMC9012716; doi:10.1007/s00267-021-01446-1)
Supplement: Supplementary file 1 — Supplementary Material [file 267_2021_1446_MOESM1_ESM.docx]

**Supplemental material**

**Operationalizing Integrated Water Resource Management in Latin America: insights from application of the Freshwater Health Index**

Maíra Ometto Bezerra, Derek Vollmer, Natalia Acero, Maria Clara Marques, Diego Restrepo, Eddy Mendoza, Bruno Coutinho, Ivo Encomenderos, Lina Zuluaga, Octavio Rodríguez, Kashif Shaad, Sarah Hauck, Ramon González, Francisco Hernandéz, Rodolfo Montelongo, Eliana Torres, Lina Serrano

**Text S1. Freshwater Health Index: Methods and Datasets**

The sections below provide an overview of the calculation process for indicators used in the manuscript and is derived from the ‘Freshwater Health Index user manual v1.2’. The authors encourage readers interested in detailed description of the methods and sample questionnaires to refer to the user manual (at:

[www.freshwaterhealthindex.org](http://www.freshwaterhealthindex.org)). Data sources for each study basin are provided following the explanation of each method.

All indicators are scaled in range 0-100.

# 1. Ecosystem Vitality Indicators

## 1.1 Water Quantity

Selected sub-indicators are intended to capture the change in stock and flows of water above and below surface. The Water Quantity is the geometric mean of two sub-indicators:

1. *Deviation from Natural Flow*

For the study in Latin America, the deviation from natural flow (DvNF) was calculated using the Amended Annual Proportion of Flow Deviation index (Gehrke et al., 1995; Gippel et al., 2011):

$AAPFD= \sum_{j=1}^{p} \frac{\sqrt[2]{\sum_{i=1}^{12} \left[ \frac{m_{i}-n_{i}}{\bar{n_{i}}} \right]^{2}}}{p}$ (1)

where, *m_i_* is monthly flow data accruing to current condition, *n_i_* is modeled natural flow for the same period. *p* is the number of years and $\bar{n_{i}}$ is mean reference flow for month *i* across *p* years.

Values are normalized to a 0-100 scale using thresholds reported in Gehrke et al. (1995):

$$DvNF= \left\{ \begin{aligned} 100-100\times AAPFD \text{for} 0\leq AAPFD<0.3 \\ 85-50\times AAPFD \text{for} 0.3\leq AAPFD<0.5 \\ 80-20\times AAPFD \text{for} 0.5 \leq AAPFD<2 \\ 50-10\times AAPFD \text{for} 2\leq AAPFD<5 \\ 0 \text{for} AAPFD \geq5 \end{aligned} \right.$$

(2)

## Current and natural discharges were modeled for all three study basins. The details for the hydrological models are provided on session five below.

## *Groundwater Depletion*

This sub-indicator measures the depletion potential of aquifers, which can be calculated from the potential depletion of the aquifers. It is calculated as the proportion of the aquifer area in the basin where depletion has been detected and the total area of the basin, according to the following formula:

$GwSD=\left( 1-\frac{\sum a}{A} \right)*100$ (3)

where, 'a' is the area where depletion problems were identified, and 'A' and the total area of the studied basin.

*Bogotá basin*: We used information from studies on the intensity of groundwater depletion in the Bogotá river basin sponsored by the Autonomous Regional Corporation of Cundinamarca (CAR) (CAR 2008). These studies provided the data on areas presenting groundwater depletion for different aquifers (Cretaceous, Quaternary and Tertiary) within the Bogotá river basin.

*Guandu basin*: Data on groundwater availability and demand was obtained from the Strategic Water Resources Plan for the Guandu, Guarda and Guandu Mirim river basins, which was available at the sub-basin level (PROFILL, 2017). Sub-basins where consumption was greater than 50% of availability were considered as areas of concern and included in the analysis.

## 1.2 Water Quality

The Water Quality Index measures how much the water quality parameters differ from the values necessary for the functioning of aquatic ecosystems and is calculated using the modified CCMW method (Saffran et al., 2001). Thresholds required for each parameter are either derived from local environmental guidelines or literature. The steps of the calculation are:

1. Calculate ‘Scope’

$F_{1}=\left( \frac{Number of failed parameters}{Total number of paramters} \right)\times100$ (4)

1. Calculate ‘Frequency & Magnitude’

For each test [i] performed for each parameter, excursion beyond threshold for failed tests is calculated as:

$\mathrm{Ex}_{i}= \left( \frac{{Failed test value}_{i}}{\mathrm{Threshold}_{i}} \right)-1$ (5)

Or,

$\mathrm{Ex}_{i}= \left( \frac{\mathrm{Threshold}_{i}}{{Failed test value}_{i}} \right)-1$ (6)

Depending if value must not exceed or fall below the threshold. The values are converted to a scale 0-100 using the following steps:

$nse=\frac{\sum_{i=0}^{n} \mathrm{Ex}_{i}}{Total number of tests}$ (7)

$F_{3}=\left( \frac{\mathrm{nse}}{nse+1} \right)\times100$ (8)

1. The F1 and F3 are combined:

$WQI=100- \sqrt{F_{1} \times F_{3}}$ (9)

*Alto Mayo basin*: Data on 21 water quality parameters (pH, dissolved oxygen, biological oxygen demand, total dissolved solids, total phosphorus, total nitrogen, nitrate, barium, selenium, antimony, arsenic, dissolved cadmium, copper, mercury, chromium, nickel, lead, thallium, zinc, oil and fats, and thermotolerant coliforms) monitored by the National Water Agency at 12 points in the Alto Mayo Basin for two years were used in the analysis (ANA, 2016, 2017). Thresholds to guarantee the stream functioning for each of those parameters were obtained from the Peruvian Regulation issued by the Ministry of the Environment (MINAM, 2017).

*Bogota basin*: Data on ten water quality parameters (total dissolved solids, dissolved oxygen, biological oxygen demand, organic nitrogen, ammonium, nitrate, organic phosphorus, orthophosphates, suspended solids, total coliforms) at 41 points along the Bogota river during the period between 2012 and 2016 were used in the analysis (CAR, 2018). Thresholds to guarantee the stream functioning for each of those parameters were obtained from the Colombian regulation (Agreement Number 43 of October 17, CAR, 2006).

*Guandu basin*: Data on six water quality parameters (pH, total phosphorus, biological oxygen demand, dissolved oxygen and temperature) monitored by the State Environmental Institute (INEA) at 28 points in the Guandu Basin during the period between 2015 and 2017 were used in the analysis (INEA, 2015, 2016, 2017a). Thresholds to guarantee the stream functioning for each of those parameters were obtained from national regulation (CONAMA, 2005).

For all three basins, average concentrations for pristine ecosystems from scientific literature (Allan and Castillo, 2007) was also used when thresholds established national regulations were considered too high to describe undisturbed conditions (e.g., concentrations of nitrate, total nitrogen, total phosphorus, and orthophosphates).

##

## 1.3 Drainage Basin Condition

The sub-indicators under this attempt to account for state of the surface waterbodies as well as landcover on freshwater health. The Drainage Basin Condition is the geometric mean of three sub-indicators:

1. *Flow connectivity*, i.e., longitudinal connectivity of stream network using Dendritic Connectivity Index (DCI)

Proposed by Cote et al. (2009), for a stream network fragmented by (n-1) impassable barriers, DCI for potamodromous and diadromous fish species are calculated as:

$DCIp=\sum_{i=1}^{n} \frac{l_{i}^{2}}{L^{2}}$ (10)

$DCId=\frac{l_{0}}{L}$ (11)

where, *L* is the total length of the river, $l_{i}$ is the length of *i*^th^ fragment, and $l_{0}$ is the length of fragment closest to the mouth of the river system.

*Alto Mayo basin*: There were no obstructions along the drainage network of the Alto Mayo basin, such as dam barriers that could prevent the free transit of fish along the drainage network. Also, no significant natural obstructions were identified in the Mayo River through recent satellite images (Google Earth, accessed October 10, 2018).

*Bogota basin*: The drainage network was developed from the 12.5-meter resolution digital terrain model of the ALOS-PALSAR satellite mission of the Japan Aerospace Exploration Agency. Drainage network modeling was preferred due to the incompatibility of the official shapefile (Bogotá, 2017). Once the complete drainage network was modeled, the stream network of the central zone of the Bogotá river basin was manually corrected to reflect the reality of the system. The location of infra-structure along the streams and rivers were obtained official sources.

*Guandu basin*: The information on the drainage network and the locations of the dams were obtained from official sources for the year of 2018 (IBGE, 2018a,b).

1. *Bank modification,* i.e., lateral connectivity of stream network using percent of channel modification (pCM)

The predominant vegetation along a fixed-distance riparian buffer (30 m riparian buffer) was used as a proxy for channel modification at the reach scale, where geomorphic adjustments to altered water and sediment regimes can have immediate consequences for stream ecosystems via changes in habitat structure and dynamics. A decision matrix relating land use with a scale from 0 to 1 (0 for near-natural, 1 for fully channelized) was then used to calculate the final score. Scores for [i] sub-basins were combined using:

$pCM=\left( 1- \frac{\sum_{i=1}^{n} l_{i}{pCM}_{i}}{L} \right)*100$ (12)

where, *L* is the river network length, $l_{i}$ is the length of the river fragment in *i*th sub-basin.

*Alto Mayo basin*: Land use along the riparian zones along the entire drainage network was extracted from official land use map (MINAM 2018). The following scores were used: 0 for natural vegetation; 0.25 for farms; and 0.5 for urban areas.

*Bogota basin*: Land use along the riparian zones along the entire drainage network was extracted from official land use map – 1:100,000 (IDEAM 2013). The following scores were used: 0 for natural vegetation; 0.25 for secondary natural vegetation, and 0.5 for land use characterized by a mosaic of agricultural crops and natural environment, and completely agricultural areas. Additionally, significant changes (channelization and artificial channels) were identified from the analysis of satellite images and received a score of 1.

*Guandu basin*: Land use along the riparian zones along the entire drainage network was extracted from official land use map for the year of 2015 (INEA, 2017). The following scores: 0 for no visible change in natural vegetation; 0.25 for areas of secondary forest and reforestation; 0.5 for agricultural areas; 0.75 for pasture areas and cleared areas; 0.85 for reservoirs; and 1 for channels in urban areas and bare soils. Significant changes (channelization and artificial channels) were identified from the analysis of satellite images and these areas received a score of 1.

1. Land cover Naturalness, i.e., amount of human-induced transformation present in land cover (LCN)

A Degree of Naturalness classification matrix is applied to each land-cover/land use (LULC) category available from the LULC map of the basin. The proposed weighting for “naturalness” in the matrix included ranges of values to help highlight transitions from “natural” to “transformed” systems, i.e., from forests and wetlands to cultivated lands or from cultivated lands to urban areas – and was prepared/refined with help of local expert opinion.

The weights for each LULC type are combined using area covered by each LULC type as multiplier.

*Alto Mayo basin*: We used the land use map from the National Forest Conservation Program for the year of 2016 (MINAM, 2018). The land cover/use types in the PNCB map received scores ranging from 0 to 100 according to the following criteria: 100 for natural vegetation cover and seminatural; 60 for the assisted cultural system (mosaic of wetlands and agriculture); 50-30 for transformed system (pastures and agriculture); and 10 for completely artificial areas, for example, urban areas.

*Bogota basin*: We used the land use map from the Environmental Information System of Colombia (IDEAM, 2013). Each land use/cover type received scores ranging from 0 to 100 according to the following criteria: 100 for natural vegetations; 90 for transformed natural environments; 70 for environments characterized by a mosaic of agricultural crops and natural environment; 40 for fully agricultural areas; and 10 to completely artificial areas, for example, urban areas.

*Guandu basin*: We used the land use map produced by the INEA for the year 2015 (INEA, 2017b). The different classes of land use on the map received scores varying from 0 to 100 according to the following criteria: 100 for natural areas and forests in advanced stages of succession, 70 for forests in the early stages of succession, 50 for commercial reforestation, 35 for agriculture, 20 for pasture, 15 for flooded areas, 10 for bare soil, since they are related to areas of sand extraction, and 0 for urban areas.

## 1.4 Biodiversity

The biodiversity indicator is the geometric mean of two sub-indicators: species of concern, and invasive and nuisance species.

Species of concern (${ISC}_{i}$) has three components (1) the proportion of threatened freshwater species ($I_{TE,i}$), (2) change in the number of species of concern (${\Delta SC}_{i}$), and (3) average population trend across all species of concern (${PT}_{i}$). These three parameters are then combined to give an overall index for the status and change in species of concern.

${ISC}_{i}=min\left\{ {ISC}_{i-1} \sqrt[3]{{I_{TE,i}\times\Delta SC}_{i}\times{PT}_{i}} , 100 \right\}$ (12)

Due to data availability constraints, only $I_{TE,i}$ was calculated and ${\Delta SC}_{i}$ or ${PT}_{i}$ were set to equal 1 for the calculation of ${ISC}_{i}$.

1. *Species of Concern*

The proportion of threatened freshwater species ($I_{TE,i}$) is calculated by determining the weighted proportion of freshwater species either as critically endangered (CR), endangered (EN), or vulnerable (VU) against the total number of species assessed (using IUCN Red list classification); calculated as:

$$I_{TE,i}=1- \frac{w_{CR}n_{CR,i}+w_{EN}n_{EN,i}+w_{VU}n_{VU,i}+\sum_{j} w_{j}n_{j,i}}{(w_{CR}n_{CR,i}+w_{EN}n_{EN,i}+w_{VU}n_{VU,i}+\sum_{j} w_{j}n_{j,i} +w_{NotT}n_{NotT})}$$

(13)

where $n_{CR,i}, n_{EN,i},$ and $n_{VU,i}$are the number of species listed as CR, EN, or VU under the IUCN Red List categories and criteria at time *t* = *i,* respectively, $n_{j,i}$ is the number of species classified in an endangered or threatened category at the national or provincial level at time *i* (e.g., for regions that classify species as “endangered” or “threatened”, *j*=1 refers to the endangered category and *j*=2 refers to the threatened category), $n_{NotT}$ refers to the remaining assessed species that are not classified in a threatened category (e.g. Least Concern [LC], or Near Threatened [NT] in the IUCN Red List), $w_{CR}, w_{EN}$, $w_{VU},$and $w_{NotT}$ are weights applied to the number of CR, EN, VU and not threatened species, respectively, $w_{j}$ are the weights applied to the number of endangered and threatened species at the national or provincial level. The sum of all $n_{x, y}$ is the total number of species assessed in the basin under the IUCN Red List criteria and/or national or provincial criteria. Weights should be assigned such that $w_{CR}\geq w_{EN}\geq w_{VU}\geq w_{NotT}$ and $w_{j}\geq w_{j+1}\geq w_{NotT}$.

For all three study basins the primary data was obtained from IUCN (2018). However, additional species data was included as following:

*Alto Mayo basin*: Bird data was obtained from the report on the Maranon-Alto Mayo Conservation Corridor (Angulo et al., 2008) and the report on Birds of the Clouds (Plenge et al., 2004). Fish data was obtained from Alessandra Escurra thesis (Escurra, 2017). In addition, a few non-aquatic "umbrella" species were included in the analysis per stakeholder suggestion, which can be justified because there is a relationship between biodiversity and habitat loss (Cardinale et al., 2012).

*Bogota basin*: No additional data was included.

*Guandu basin*: IUCN’s data list was complemented with species data from the Strategic Water Resources Plan for the Guandu River Basin (PROFILL, 2017). Fish data was obtained from the Identification Guide of the Rio das Pedras Basin, Rio Claro municipality (Buckup et al., 2014)

1. *Invasive and nuisance species (*${INS}_{i}$*)*

It also has three components mirroring ${ISC}_{i}$ ; and only the first component: the number (i.e. richness) of invasive and nuisance species ($I_{IN,i}$), was calculated based on available data.

$I_{IN,i}= \left\{ \begin{aligned} 1-\frac{n_{IN,i}}{10}, \\ 0.1, \text{for }n_{IN,i}\geq9 \end{aligned} \right. \text{for} 0\leq n_{IN,i}\leq8$ (14)

where $n_{IN,i}$ is the number of invasive and nuisance species in the basin at time *t* = *i*.

*Alto Mayo basin*: Data on invasive species was obtained from permits for aquaculture from the Regional Production Office of the San Martin region (DIREPRO-SM, 2018).

*Bogota basin*: Data on invasive species was obtained from a report from the Corporación Autónoma Regional de Cundinamarca (Mora-Goyes and Barrera-Cataño, 2015).

*Guandu basin*: Data on invasive species was obtained from two reports (PROFILL, 2017; Buckup et al., 2014).

# 2. Ecosystem Services Indicator

## 2.1 Provisioning and Regulating services framework

This category of indicators attempts to measure the impact of Ecosystem services by considering the gap between the supply and demand of services generally associated with freshwater ecosystems. To begin, the basin is divided into spatial units or SUs (generally sub-basins or administrative units) and the supply-demand gap is evaluated over each SU. ‘Failure’ in this case is: inability of supply to meet demand.

The steps of the calculation are:

a) Calculate ‘Scope’

$F_{1}=\left( \frac{No. of SU failed}{Total number of SU} \right)\times100$ (15)

b) If data on number of times (instances) failure occurs is available, then calculate ‘Frequency’

$F_{2}= \left( \frac{Number of instances failed}{Total number of instances} \right) \times100$ (16)

c) If information on scale of failure is available, then calculate ‘Frequency & Magnitude’

For each time step [i] for each SU, excursion beyond threshold for failed instances is calculated as:

$\mathrm{Ex}_{i}= \left( \frac{{Failed instance value}_{i}}{\mathrm{Threshold}_{i}} \right)-1$ (17)

Or,

$\mathrm{Ex}_{i}= \left( \frac{\mathrm{Threshold}_{i}}{{Failed instance value}_{i}} \right)-1$ (18)

Depending if value must not exceed or fall below the threshold. The values are converted to a scale 0-100 using the following steps:

$nse=\frac{\sum_{i=0}^{n} \mathrm{Ex}_{i}}{Total number of instances}$ (19)

$F_{3}=\left( \frac{\mathrm{nse}}{nse+1} \right)\times100$ (20)

1. Based on availability of data, combine values to derive score:

- If able to only determine F1: $ESI=100 - F1$ (low evidence)
- If able to only determine F1 and F2: $ESI = 100 - \sqrt{F1\times F2}$ (medium evidence)
- If able to determine all three: $ESI = 100 - \sqrt{F1\times F3}$ (high evidence)

(21)

*a) Water Supply Reliability Relative to Demand*

*Alto Mayo basin*: We used data on annual water allocation (proxy for water demand) for different sectors and annual cumulative volume of surface runoff from the “Assessment and Accounting of Ecosystem Values” (EVA) study for the San Marín region in Peru (CI, 2016). Data was specific for the Alto Mayo basin and included three years (2009, 2011 and 2013). The objective aim at meeting 100% of the demand of each sector in each year.

*Bogotá basin*: Data on water supply and demand for 3rd-order sub-basins within the Bogota basin was obtained from the Regional Water Studies (ERAs) (CAR, 2017a,b,c,d). From this data the potential water supply deficit for each sub-basin was estimated with an objective of meeting a 100% of the demand.

*Guandu basin*: 2017 water availability and demand data from six sectors (urban supply, rural supply, industry, thermoelectric, irrigation, mining, and animal watering) related to 13 hydrological administrative units - HAU (PROFILL, 2017). It was assumed that the maximum water availability to meet the demands in each HAU was 50% of Q7,10 of the total water volume in each respective HAU, which is the same criterion adopted by INEA for funding grants. The objective aim at meeting 100% of the demands of each sector within each HAU.

*b) Sediment Regulation*

*Alto Mayo basin*: The erosion rates estimated were modeled for eight sub-basins for two years (2011 and 2013) using the USPED (Unit Stream Power based Erosion Deposition, Mitasova et al., 1996; Mitas and Mitasova,1998). For each year, the average erosion rate in deforested areas within each sub-basin was derived. Since the erosion model was not calibrated and validated, it was not possible to use tolerable erosion values described in the scientific literature as thresholds. Therefore, thresholds were derived from the modeling itself, i.e., the average erosion rate observed in areas covered by natural vegetation within the entire Alto Mayo basin was estimated to be used as the objective to be met.

*Bogota basin*: We used the erosion map, “Soil Zoning by Degree of Erosion, Baseline 2010-2011” (IDEAM, 2015), from which eroded areas in each of the municipalities within the Bogota basin were defined. The selected threshold corresponded to a maximum of 20% of the total area of the municipality with erosion.

*Guandu basin*: Data on total dissolved solids and turbidity monitored by the State Environmental Institute at 28 points in the Guandu Basin during the period between 2015 and 2017 were used in the analysis (INEA, 2015,2016,2017). Thresholds to guarantee water potability each parameter were obtained from national regulation (CONAMA, 2005).

*c) Water Quality Regulation*

The same water quality data used to calculate the indicator of water quality in the Ecosystem Vitality component was used to calculate this indicator for each basin. The difference is that for Water Quality Regulation the threshold was considered those associated with each specific water use defined for different stream segments.

*Alto Mayo basin*: Thresholds were obtained from national regulation (MINAM, 2017) and included values for Category 1, Subcategory A2.

*Bogota basin*: Thresholds were obtained from national regulation (CAR, 2006) and included values for the five different sessions the basin was been divided.

*Guandu basin*: Thresholds were obtained from national regulation (CONAMA, 2015) and included values for Special Class, Class 1 and Class 2.

*d) Disease Regulation*

Each country included more than one disease in the analysis. For each disease, an individual score was calculated, and the final score was the geometric mean obtained considering the values for each disease.

*Alto Mayo basin*: We used data on the occurrence of four water-related diseases (dengue, zika, chikungunya and malaria) in 15 districts of the provinces of Rioja and Moyobamba (Calzada, Habana, Jepelacio, Moyobamba , Soritor, Yantaló, Awajún, Nueva Cajamarca, Pardo Miguel, Posic, Rioja, San Fernando, Yorongos, Yuracyacu) during the last three years (2015-2017) (CDC, 2018). The analysis also included fecal coliform data as a proxy for the occurrence of diarrhea that may be related to water.

*Guandu basin*: We used data on the occurrence of three water-related diseases (dengue, yellow fever and malaria) in 13 municipalities within the Guandu Basin (Seropedica, Eng. Paulo de Frontin, Itaguai, Japeri, Miguel Pereira, Paracambi , Burns, Rio Claro, Pirai, Mangaratiba, Rio de Janeiro, Nova Iguacu, Mendes) during 2015, 2016 and 2017 (DATASUS, 2018a,b,c). The municipalities of Barra do Piraí and Vassouras were not included in the analysis because only a very small part of their area is within the basin. Disease thresholds were established by regulatory review and world literature detailed below. The analysis also included fecal coliform data as an approximation of the occurrence of diarrhea that may be related to water.

The same set of thresholds were used for Alto Mayo and Guandu. The thresholds were established based on review of regulations and literature related to what would be considered a "normal" occurrence rate of a particular disease in a given population. For each of the diseases, an annual outbreak rate was found. In the case of dengue, zika and chikungunya, the annual outbreak rate considered was three occurrences for every 1000 people (Regau-Perez et al., 1999) and the outbreak rate for malaria was one occurrence for every 100 people (PAHO/WHO, 2016). For fecal coliforms, the thresholds were from the water regulations determining the maximum allowed concentration for drinking water determined by each country.

*e) Flood Regulation*

*Alto Mayo basin*: We used data on the number of people affected by floods in 15 districts of the provinces of Rioja and Moyobamba (Calzada, Habana, Jepelacio, Moyobamba, Soritor, Yantaló, Awajún, Nueva Cajamarca, Pardo Miguel, Posic , Rioja, San Fernando, Yorongos, Yuracyacu) for the period from 2015 to 2017 (SINPAD, 2018). The threshold was assumed to be zero.

*Bogota basin*: Spatially explicit data on floods over the entire territory of the CAR jurisdiction was used. The threshold was assumed to be zero.

*Guandu basin*: We used data on occurrence of leptospirosis in 13 municipalities for the period 2015-2017 (DATASUS, 2018d). The Brazil’s Ministry of Health considers that leptospirosis is an infectious disease transmitted to humans by rodent urine, especially during floods, thus assumed as a good proxy to calculate this indicator. For the threshold, a value of zero was assumed, as flooding with direct human impacts should not occur.

## 2.2 Cultural Services

The two dimensions for cultural services that can be measured are (1) Conservation & Cultural Heritage; and (2) Recreation. For the three study basins. Available data allowed the calculation only of the former.

For each basin, Conservation & Cultural Heritage was estimated from maps of coverage showing protected areas (PAs) and the drainage network. The river length within the protected areas system and length of streams that formed protected area boundaries was determined from the river network dataset. These were compared against the total river length within each study basin with the following formula:

$PoR= \frac{0.5 * BL+IL}{RL}*100$ (22)

where PoR is the percent river length protected; BL, the length of rivers bordering protected areas; IL, the length of rivers within protected areas; and RL is the total length of rivers within the study basin.

With the global target of minimum wetlands and waterways under protected areas set at 17% under the Convention on Biological Diversity Aichi Biodiversity Target 11 (https://www.cbd.int/sp/targets/), the value was scaled using an asymptotic function:

$CS=117 \frac{PoR}{Por+17}$ (23)

where CS is the score of the sub-indicator Conservation & Cultural Heritage and PoR is the percent river length protected.

*Alto Mayo*: Conservation areas were mapped according to three different official sources (IBC, 2016; IDERSAM, 2018; SERNANP, 2018) and the drainage network data according to the Instituto Geográfico Nacional (IGN, 2018).

*Bogota basin*: Conservation areas were mapped according to the following the Parques Nacionales Naturales de Colombia (PNN, 2018) and the drainage network data according to the official source (Bogotá, 2017) that was corrected and adjusted.

*Guandu basin*: Conservation areas were mapped according to the following the Instituto Estadual do Ambiente (INEA, 2017c; INEA, 2018) and the drainage network data according to the Brazilian Institute of Geography and Statistics (IBGE, 2018a).

# 3. Governance & Stakeholder survey

The Governance & Stakeholders indicators are based on stakeholders’ perceptions and were assessed using a questionnaire consisting of 12 modules corresponding to each sub-indicator, 3-6 questions per module. A total of 49 questions were asked, each using a 1-5 Likert-type scale to quantify the qualitative responses. Responses were consistently phrased so that higher scores on the scale correspond to a more positive assessment. For example, the five questions pertaining to “Water-Related Conflict” use a scale where 1 = Conflicts almost always occur and 5 = Conflicts almost never occur. The mean value for each response was used to calculate final (sub) indicator scores.

# 4. Indicator weights using AHP

To ensure that aggregated indicator values for both Ecosystem Services and Governance & Stakeholders reflected stakeholders’ preference, stakeholders are surveyed to complete a weighting exercise based on the Analytic Hierarchy Process (Saaty, 2005). A hierarchy was created so that stakeholders made a total of 34 pairwise comparisons, first amongst major indicators in each component, and then amongst sub-indicators within a major indicator category. The stakeholders completed the exercise, first by selecting the (sub) indicator they considered more important, and then rating how much more important using a 1-9 intensity scale (where 1 was used to indicate “no preference” between the two objects being compared). These numeric scores were translated into a reciprocal matrix and the principal right eigenvector was calculated to derive weights between 0 and 1. The BPMSG AHP Online System (Goepel, 2013) was used to design, administer (in English), and process the exercise. The mean group value was used for weighting aggregated indicators, though individuals’ consistency ratios (CR) and the strength of consensus for each choice task are also evaluated.

**5. Hydrological models for DvNF**

The AAPFD was calculated with estimated data representing the estimated flows for natural conditions and for flows representing current conditions. The AquaTool program (Andreu et al., 1996) was used with a hydrological modeling platform to estimate two flows expected in natural conditions. More specifically, the EVALHID module was used, which is a module for the development of rain-flow models in complex basins and that evaluates the quantity of water resources produced. Within EVALHID, the Témez model (Temez, 1997) was used to estimate the surface flow of rivers under natural conditions.

*Alto Mayo basin*: The hydrological model included eight sub-basins and was carried out for the period from January 1998 to December 2017. The flows for natural conditions were simulated considering the vegetation cover map available for the study area (MINAM, 2015). Daily rainfall and temperature data was obtained from 16 climatological stations administered by different government institutions (Autoridad Local del Agua, ALA; Autoridad Nacional del Agua, ANA; Proyecto Especial Alto Mayo, PEAM; and Servicio Nacional de Meteorología e Hidrología del Perú, SENAMHI) that covered the entire study area for a period of 20 years (1998 to 2017). As the time series presented missing data, satellite information from the GIOVANNI database (NASA, 2018) was used as a complement. As input data itself, the model used monthly precipitation values, which were obtained using the Thiessen polygon method (Thiessen, 1911). Temperature data were used to estimate potential evapotranspiration by the Thornwaite method (Thornwhite, 1948). The required flow data were obtained from eight flow measurement stations administered by different government institutions (ALA, ANA, SENAMHI) with data for a period of 11 years (2001-2011). The calibration period of the model was from 2001 to 2011.

*Bogota basin*: The hydrological modeling was carried out for a smaller area of the Bogota basin (Figure S3), upper region, as this is a strategic water source area to the metropolitan city of Bogota. Input data included: monthly precipitation and temperature data from 65 weather stations (CAR, and IDEAM) covering the entire study area for the period from January 1994 to December 2015. Because these data series had missing data, we used two methods to complete the time series: 1) rational deductive method when the gaps in the monthly data were not for the entire years (Campos, 1998) and 2) inverse squared distance method (Chow et al., 1996) when an entire year had no data. The precipitation and average temperature for each of the sub-basins was calculated using the method of the Thiessen polygons (Thiessen, 1911). Potential evapotranspiration data were estimated for the area using the Thornwaite method (Thornwhite, 1948) from monthly temperature data. Discharge data was obtained from six gauging stations administered by CAR with data for the period from 2005 to 2015.

*Guandu basin*: The hydrological modeling included 17 sub-basins and was performed for the period from January 2004 to December 2016. The natural flows were simulated considering the potential natural vegetation map available for the region (INEA, 2010). Monthly precipitation data from 14 climatological stations administered by different institutions (Agência Nacional de Águas, ANA; Instituto Estadual do Ambiente, INEA; Light Serviços de Eletricidade S.A, LIGHT) were used covering the entire study area from January 1990 to March 2018. Temperature data were obtained from eight stations administered by different government institutions (Fundação Instituto de Geotécnica, GEORIO; Instituto Nacional de Meteorologia, INMET) for the same period. The precipitation and average temperature for each of the sub-basins were calculated using the method of the Thiessen polygons (Thiessen, 1911). Missing data were estimated by doing an arithmetic mean of the records in the surrounding stations. In other cases, an adjustment was made based on meteorological information downloaded from the GIOVANNI interface (Acker and Leptouckh, 2007) using the Tropical Rainfall Measuring Mission (TRMM) model, this strategy was used to fill incomplete data sets for short periods of time for some climatological and pluviometric stations. Potential evapotranspiration data were estimated for the area using the Thornwaite method (Thornwhite, 1948) from monthly temperature data. In total, flow data from 18 flow measurement stations were used. The calibration of the model considered three main sub-basins identified in the area, the contribution and volume of surface water, as well as the transfer volumes. The three basins used for calibration were: 1) Lajes Tocos-Dam transfer system, as well as the contribution to the Tocos dam. The calibration period for this section was from 2003 to 2018; 2) transfer of the Lajes dam system with the contribution of the Ribeirão das Lajes basin and the Fontes-Lajes dam model and its contribution to the UHE Fontes Nova hydroelectric plant. The calibration period for this section was from 2003 to 2018; and 3) Santana-Vigario system, which receives flow from the Piraí basin and contribution from the transfer of the Paraíba do Sul river basin. The calibration period for this section was from 2003 to 2017.

**
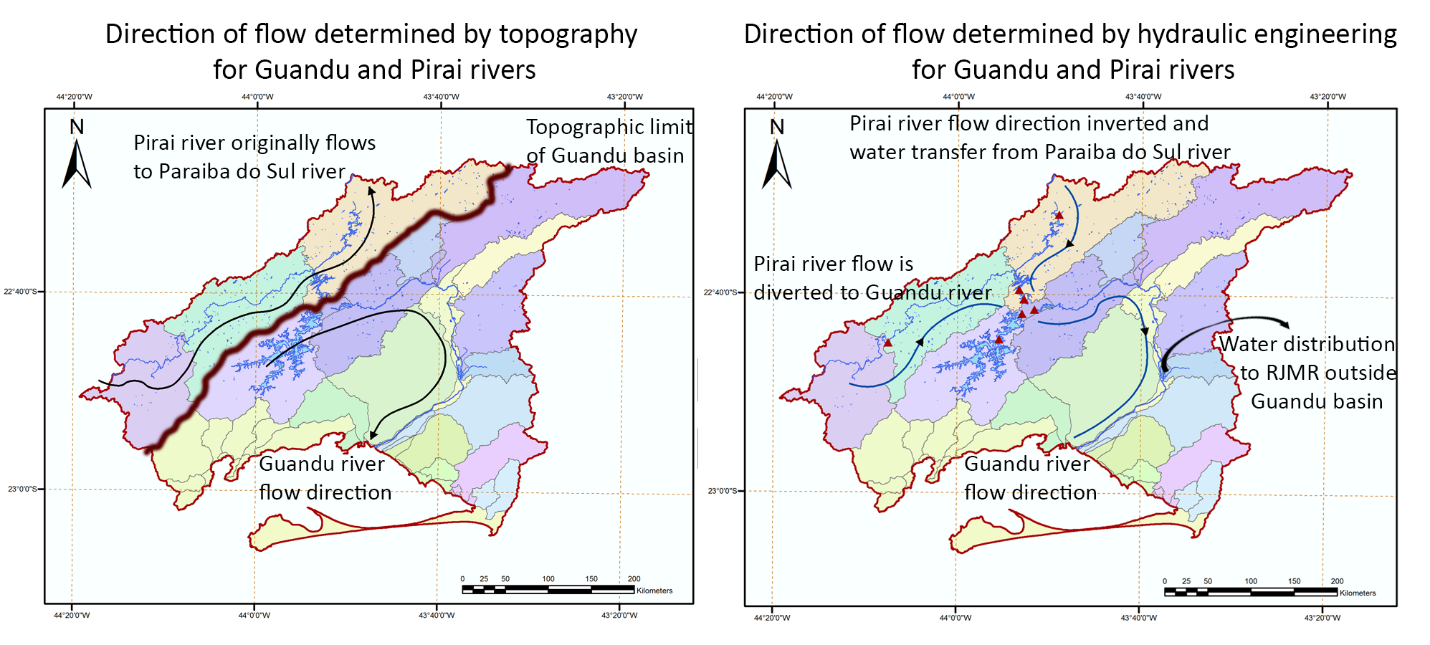
**

**Figure S1.** Flow direction of major rivers of the Guandu River basin based on topography (left), and hydraulic engineering (right). Red triangles are major reservoirs

**Figure S2**. Box plots for sensitivity analyses of AHP-derived weights. Graphs represent the range of potential scores (component, followed by major indicators) for both Ecosystem Services and Governance & Stakeholders in Guandu (a), Alto Mayo (b), and Bogotá (c) respectively. Note that the scales differ between the ES and GS graphs. And, since Provisioning and Cultural Services indicators only included one measured sub-indicator each, we did not perform a sensitivity analysis on them but include them as constants (the actual measured score) when assessing the sensitivity on the ES component.

**
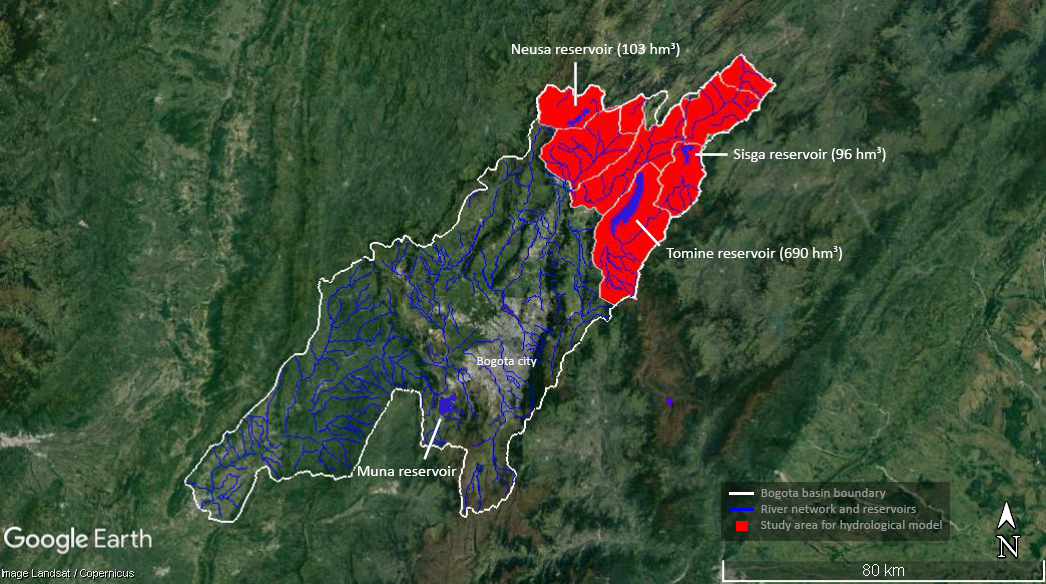
**

**Figure S3.** Hydrological subzone (specific study area) used for the indicator of Deviation of Natural Flow for the Bogota River basin. Main reservoirs in the study area are highlighted with their water holding capacity in parenthesis

**Additional supplementary references**

Acker, J.G., Leptoukh, G., 2007. Online Analysis Enhances Use of NASA Earth Science Data. Eos, Transactions AGU 88(2): 14-17. https://doi.org/10.1029/2007EO020003

Allan, J. D., Castillo, M.M., 2007. Nutrient dynamics. In J.D. Allan and M.M. Castillo (Eds), Stream Ecology: Structure and Function of Running Waters (pp. 255-286). 2nd Edition. New York, NY: Chapman and Hall.

Andreu, J., Capilla, J., Sanchis, E., 1996. AQUATOOL, a generalized decision support system for water resources planning and operational management. Journal of Hydrology 177(3-4): 269-291.

Angulo, P., Palomino, W., Arnal, H., Aucca, C., Uchofen, O., 2008. Corredor de conservación de aves Marañón - Alto Mayo: análisis de distribución de aves de alta prioridad de conservación e identificación de propuestas de áreas para su conservación. Asociación Ecosistemas Andinos (ECOAN) and American Bird Conservancy (ABC). Cusco, Perú, 147p.

Autoridad Nacional del Agua (ANA), 2016. Informe de monitoreo participativo de la calidad de agua superficial de la cuenca del río Huallaga.

Autoridad Nacional del Agua (ANA), 2017. Informe de monitoreo participativo de la calidad de agua superficial de la cuenca del río Huallaga.

Buckup, P. A., Britto, M. R., Souza-Lima, R., Pascoli, J. C., Villa-Verde, L., Ferraro, G. A., Salgado, F. L. K., Gomes, J. R., 2014. Guia de identificação das espécies de peixes da bacia do rio das Pedras, município de Rio Claro, RJ. 2014. Rio de Janeiro, RJ: The Nature Conservancy.

Bogotá, DC, 2018. Datos abiertos Bogotá. Corriente de Agua. Bogotá D.C. Retrieved from https://datosabiertos.bogota.gov.co/dataset/corriente-de-agua-bogota-d-c

Campos, D.F., 1998. Procesos del ciclo hidrológico. San Luis Potosí: Universidad Autónoma de San Luis Potosí (UASLP).

Cardinale, B.J., Duffy, E., Gonzalez, A. Hooper, D.U., Perrings, C., Venail, P., Narwani, A., Mace, G.M., Tilman, D., Wardle, D.A., Kinzig, A.P., Daily, G.C., Loreau, M., Grace, J.B., Larigauderie, A., Srivastava, D., Naeem, S., 2012. Biodiversity loss and its impact on humanity. Nature 486(7401): 59-67. http://dx.doi.org/doi:10.1038/nature11148.

Centro Nacional de Epidemiología, Prevención y Control de Enfermedades (CDC), 2018. Sala situacional. Lima, Perú. Retrieved from <http://www.dge.gob.pe/salasituacional/sala/index/eventos/128>.

Chow, V.T., Maidment, D.R., Mays, L.W., 1996. Hidrología aplicada. Bogotá: McGraw Hill.

Conselho Nacional do Meio Ambiente (CONAMA), 2005. Resolução no 357, de 17 de março de 2005. Retrieved from <http://www2.mma.gov.br/port/conama/legiabre.cfm?codlegi=459>.

Conservation Internacional Perú (CI), 2016. Cuentas Experimentales de los Ecosistemas en San Martin – Perú: Conservation International. 62p.

Corporación Autónoma Regional de Cundinamarca (CAR), 2006. Acuerdo 43 Calidad del Agua en Cuenca del Río Bogotá/2020-2006.

Corporación Autónoma Regional de Cundinamarca (CAR), 2008. Plan de manejo ambiental de agua subterránea en la Sabana de Bogotá y Zona Crítica. Bogotá.

Corporación Autónoma Regional de Cundinamarca (CAR), 2017a. Evaluación regional del Agua- ERA, Cuenca Alta Río Bogotá.

Corporación Autónoma Regional de Cundinamarca (CAR), 2017b. Evaluación regional del Agua- ERA, Cuenca Media Río Bogotá.

Corporación Autónoma Regional de Cundinamarca (CAR), 2017c. Evaluación regional del Agua- ERA, Cuenca Sumapaz.

Corporación Autónoma Regional de Cundinamarca (CAR), 2017d. Evaluación regional del Agua- ERA, Cuenca Alta Río Bogotá. 2017.

Corporación Autónoma Regional de Cundinamarca (CAR), 2018. Boletines de calidad hídrica [2012-18]. Retrieved from https://www.car.gov.co/vercontenido/2314.

Departamento de Informática do Sistema Único de Saúde (DATASUS), 2018a. Dengue – Notificações Registradas no Sistema de Informação de Agravos de Notificação – Rio de Janeiro. [Data File]. Retrieved from http://tabnet.datasus.gov.br/cgi/deftohtm.exe?sinannet/cnv/denguebrj.def.

Departamento de Informática do Sistema Único de Saúde (DATASUS), 2018b. Febre Amarela – Notificações Registradas no Sistema de Informação de Agravos de Notificação – Rio de Janeiro. [Data File]. Retrieved from http://tabnet.datasus.gov.br/cgi/deftohtm.exe?sinannet/cnv/febreamarelarj.def

Departamento de Informática do Sistema Único de Saúde (DATASUS), 2018c. Malária – Notificações Registradas no Sistema de Informação de Agravos de Notificação – Rio de Janeiro. [Data File]. Retrieved from <http://tabnet.datasus.gov.br/cgi/deftohtm.exe?sinannet/cnv/malarj.def>.

Departamento de Informática do Sistema Único de Saúde, 2018. Leptospirose – Notificações Registradas no Sistema de Informação de Agravos de Notificação – Rio de Janeiro. [Data File]. Retrieved from http://tabnet.datasus.gov.br/cgi/deftohtm.exe?sinannet/cnv/leptorj.def.

Dirección Regional de la Producción, San Martin (DIREPRO-SM), 2018. Autorización para desarrollar la actividad de acuicultura de recursos limitados.

Escurra, A., 2017. Diversidad ictiológica y estado de conservación del río Mayo (provincias de Rioja, Moyobamba y Lamas), cuenca del río Huallaga, San Martín (2006 – 2017). Unpublished undergraduate thesis. Universidad Nacional Mayor de San Marcos. Lima, Perú.

Gehrke, P., P. Brown, C.B. Schiller, D.B. Moffatt, Bruce, A., 1995. River regulation and fish communities in the Murray Darling river system, Australia. Regulated Rivers: Research& Management 15:181-198. <https://doi.org/10.1002/rrr.3450110310>.

Geoportal del Gobierno Regional de San Martin (IDERSAM), 2018. Zonas de conservación y recuperación de ecossistemas (ZOCres). Gobierno Regional de San Martin. Retrieved from https://geoportal.regionsanmartin.gob.pe/visor/.

Gippel, C. J., Y. Zhang, X. Qu, W. Kong, N. R. Bond, X. Jiang, Liu, W., 2011. River Health Assessment in China: Comparison and Development of Indicators of Hydrological Health. Brisbane.

Instituto de Hidrología, Meteorología y Estudios Ambientales (IDEAM), 2015. Zonificación de la degradación de suelos por erosión. Línea base 2010 - 2011. Sistema de Información Ambiental de Colombia.

Instituto de Hidrología, Meteorología y Estudios Ambientales (IDEAM), 2013. Mapa de cambio de bosque 2012- 2013 para Colombia. Sistema de Información Ambiental de Colombia.

Instituto Brasileiro de Geografia e Estatística (IBGE), 2018a. Base Cartográfica Vetorial Contínua do Estado do Rio de Janeiro na Escala 1:25.000 – Trecho de Drenagem. [Shapefile]. Retrieved from <ftp://geoftp.ibge.gov.br/cartas_e_mapas/bases_cartograficas_continuas/bc25/rj/versao2018/shapefiles/>.

Instituto Brasileiro de Geografia e Estatística (IBGE), 2018b. Base Cartográfica Vetorial Contínua do Estado do Rio de Janeiro na Escala 1:25.000 – Barragem. [Shapefile]. Retrieved from <ftp://geoftp.ibge.gov.br/cartas_e_mapas/bases_cartograficas_continuas/bc25/rj/versao2018/shapefiles/>.

Instituto del Bien Común (IBC), 2016. Unidades de conservación. Sistema de Información sobre comunidades nativas de la Amazonia peruana (SICNA). Retrieved from http://www.ibcperu.org/mapas/sicna/.

Instituto Estadual do Ambiente (INEA), 2010. Vegetação Potencial do Estado do Rio de Janeiro na Escala 1:100.000. [Shapefile]. Retrieved from https://inea.maps.arcgis.com/apps/MapSeries/index.html?appid=00cc256c620a4393b3d04d2c34acd9ed.

Instituto Estadual do Ambiente (INEA), 2015. Boletim consolidado de qualidade das águas da região hidrográfica II - Guandu 2015. Histórico 2015. Governo do Estado do Rio de Janeiro. Retrieved from <http://www.inea.rj.gov.br/ar-agua-e-solo/qualidade-das-aguas-por-regiao-hidrografica-rhs/>

Instituto Estadual do Ambiente (INEA), 2016. Boletim consolidado de qualidade das águas da região hidrográfica II - Guandu 2016. Histórico 2016. Governo do Estado do Rio de Janeiro. Retrieved from <http://www.inea.rj.gov.br/ar-agua-e-solo/qualidade-das-aguas-por-regiao-hidrografica-rhs/>

Instituto Estadual do Ambiente (INEA), 2017a. Boletim consolidado de qualidade das águas da região hidrográfica II - Guandu 2017. Histórico 2017. Governo do Estado do Rio de Janeiro. Retrieved from <http://www.inea.rj.gov.br/ar-agua-e-solo/qualidade-das-aguas-por-regiao-hidrografica-rhs/>

Instituto Estadual do Ambiente (INEA), 2017b. Mapeamento do uso e cobertura do solo do Estado do Rio de Janeiro no ano de 2015 na escala 1:100,000. Retrieved from <https://inea.maps.arcgis.com/apps/MapSeries/index.html?appid=00cc256c620a4393b3d04d2c34acd9ed>.

Instituto Estadual do Ambiente (INEA), (2017c). Mapa das Unidades de Conservação Federais. Retrieved from: <https://inea.maps.arcgis.com/apps/MapSeries/index.html?appid=00cc256c620a4393b3d04d2c34acd9ed>

Instituto Estadual do Ambiente (INEA), (2018). Mapa das Unidades de Conservação Federais. Retrieved from: <https://inea.maps.arcgis.com/apps/MapSeries/index.html?appid=00cc256c620a4393b3d04d2c34acd9ed>

Instituto Geográfico Nacional (IGN), 2018. Hidrografia. Información Geoespacial Fundamental. Centro de Infraestructura de Datos Geoespaciales. Geovisor de Datos Fundamentales. Retrieved from https://www.idep.gob.pe/geovisor/DatosFundamentales/.

International Union for Conservation of Nature (IUCN), 2018. The IUCN Red List of Threatened Species.

Mitas, L. and Mitasova, H., 1998. Distributed soil erosion simulation for effective erosion prevention. Water Resources Research 34(3): 505-516. https://doi.org/10.1029/97WR03347.

Mitasova, H., Hofierka, J., Zlocha, M., Iverson, L., 1996. Modeling topographic potential for erosion and deposition using GIS. International Journal of GIS 10(5): 629-641. https://doi.org/10.1080/02693799608902101.

Ministerio del Ambiente (MINAM), 2015. Mapa nacional de cobertura vegetal: memoria descriptive. Ministerio del Ambiente, Dirección General de Evaluación, Valoración y Financiamiento del Patrimonio Natural. Lima: MINAM.

Ministerio del Ambiente (MINAM), 2017. Decreto Supremo N° 004-2017. Publicado en el diario El Peruano, 07 de junio de 2017.

Ministerio del Ambiente (MINAM), 2018. Mapa de uso y cambio de uso de la tierra de la Amazonia peruana (escala 1:100,000). Programa Nacional de Conservación de Bosques (PNCB). Plataforma de monitoreo de cambios sobre la cobertura de los bosques - GeoBosques. Retrieved from <http://geobosques.minam.gob.pe/geobosque/view/descargas.php>.

Mora-Goyes M.F., Barrera-Cataño, J.I., 2015. Catálogo de especies invasoras del territorio CAR. Pontificia Universidad Javeriana, Corporación Autónoma Regional de Cundinamarca – CAR. Bogotá, D.C. 220p.

NASA, 2018. Earth Data. Retrieved from Giovanni: <https://giovanni.gsfc.nasa.gov/giovanni/>

Parques Nacionales Naturales de Colombia (PNN), (2018). Mapa de Parques Nacionales Naturales de Colombia. Sistema Nacional de Áreas Protegidas SINAP. Retrieved from https://www.parquesnacionales.gov.co/portal/es/mapa-de-parques-nacionales-naturales-de-colombia/

Pan American Health Organization (PAHO)/World Health Organization (WHO), 2016. Malaria in the region of the Americas by annual parasite incidence (API)-2016. Annual Country Reports to PAHO/CHA/VT/Malaria. Pan American Health Organization (PAHO)/World Health Organization (WHO). Retrieved from <https://www.paho.org/hq/images/stories/AD/CHA/malaria/malaria-americas-api-2016-web.jpg?ua=1>.

Plenge, H., Williams, R., Valqui, T., 2004. Aves de las Nubes. Perú: Alto Mayo & Cordillera de Colán - Perú. 104p.

Profill Engenharia e Ambiente (PROFILL), 2017. Plano estratégico de recursos hídricos das bacias hidrográficas dos rios Guandu, da Guarda e Guandu-Mirim. Diagnóstico – Tomo I. Relatório Técnico (AGVP_GUANDU_PRHRP02_TOMOI_R04.docx). Rio de Janeiro, Brasil: Ministério do Meio Ambiente, Agência Nacional de Águas and Sondotécnica. 554p.

Regau-Perez, J.G., Millard, P.S., Walker, D.R., Deseda, C.C., Casta-Vélez, A., 1999. A deviation bar chart for detecting dengue outbreaks in Puerto Rico. American Journal of Public Health 89(3): 374-378.

Saaty, T.L., 1990. How to make a decision: the analytic hierarchy process. European journal of operational research 48(1): 9-26.

Saffran, K., K. Cash, Hallard, K., 2001. CCME Water Quality Index 1.0 User’s Manual. Canadian Water Quality Guidelines for the Protection of Aquatic Life, 1–5. Retrieved from http://www.ccme.ca/files/Resources/calculators/WQI User’s Manual (en).pdf.

Servicio Nacional de Áreas Naturales Protegidas por el Estado (SERNANP), 2018. Areas naturales protegidas a nivel nacional, regional y las areas de conservación privadas. Geoservidor. Retrieved from <http://geo.sernanp.gob.pe/geoserver/principal.php>.

Sistema de Información Nacional para la Respuesta y Rehabilitación (SINPAD), 2018. Emergencias por departamento. Retrieved from http://sinpad.indeci.gob.pe/sinpad-js/.

Témez, J., 1977. Modelo matemático de transformación precipitación aportación. ASINEL.

Thiessen, A.H., 1911. Precipitation averages for large areas. Monthly Weather Review: 1082-184. https://doi.org/10.1175/1520-0493(1911)39<1082b:PAFLA>2.0.CO;2.

Thornwhite, C., 1948. An approach toward a rational classification of climate. American Geographical Society 38 (1): 55-94.
